# UV-B-induced DNA repair mechanisms and their effects on mutagenesis and culturability in *Escherichia coli*

**DOI:** 10.1128/msystems.00396-26

**Published:** 2026-06-12

**Authors:** Sreyashi Ghosh, Jenet Narzary, Mehmet A. Orman

**Affiliations:** 1 Department of Chemical and Biomolecular Engineering, University of Houston 684748, Houston, Texas, USA; 2 Department of Biomedical Engineering, University of Wisconsin-Madison 5228, Madison, Wisconsin, USA; University of Nebraska-Lincoln, Lincoln, Nebraska, USA

**Keywords:** ultraviolet-B, SOS response, mutagenesis, DNA repair, colony-forming ability, *Escherichia coli*

## Abstract

Ultraviolet-B (UV-B) radiation, intensified by ozone depletion, induces DNA damage and promotes mutagenesis, shaping evolution. While UV-induced SOS responses are well characterized in bacteria, the cellular consequences of prolonged UV-B exposure remain less clear. Prolonged UV-B exposure disrupts transcription and translation, as well as RecA-mediated SOS induction, without major changes in membrane permeability or reactive oxygen species. This impairs mutagenesis and leads to an early loss of culturability. Genetic analysis reveals both redundant and differential roles for DNA repair pathways in which homologous recombination (RecA, RecB), nucleotide excision repair (UvrA), and translesion synthesis (UmuC/D) are essential for maintaining mutagenesis and culturability, while others (RecN, RmuC) have limited impact. Notably, deletion of UvrD (a repair-associated helicase) exacerbates the early impairment in culturability without affecting mutagenesis, underscoring the complexity of repair networks. Overall, our findings reveal a dose-dependent trade-off in which moderate UV-B promotes mutagenesis with minimal viability loss, whereas prolonged exposure suppresses mutagenesis with an early impairment in colony-forming ability, reflecting an adaptive strategy with significant evolutionary implications.

**IMPORTANCE:**

Ultraviolet radiation, especially UV-B, has shaped microbial evolution for billions of years by generating DNA lesions that activate mutagenic repair pathways. Because it induces both damage and adaptive responses, UV-B serves as a powerful model for examining how environmental stress influences genetic variation. While UV exposure is known to induce SOS-mediated DNA repair, its dual effects on mutagenesis and colony-forming ability are highly interconnected and complex, requiring further mechanistic analysis. This study provides insight into how prolonged UV-B exposure modulates these pathways, linking genetic responses with shifts in cellular states that influence culturability and mutagenic potential. Collectively, these findings can be highly informative for fundamental studies of microbial adaptation and for practical applications, where UV-B is used as a selective or perturbative tool, including biotechnology, directed evolution, and disinfection strategies.

## INTRODUCTION

Mutagenic processes are key to evolutionary progress (1–3), as they generate genetic diversity. This is essential for organisms to adapt to and endure in dynamic settings that are continuously changing. Understanding the mechanisms underlying mutagenesis helps us better comprehend the evolutionary forces shaping organisms (4, 5). This knowledge also aids in developing more potent and effective medical treatments to combat many diseases, given that pathogenic microorganisms and tumorigenic cells develop resistance through mutagenesis (6–9).

Ultraviolet (UV) radiation, particularly UV-B, plays a dual role in Earth’s ecosystems; it acts as both a developmental cue and an environmental stressor. While moderate UV-B levels may regulate cellular defense and repair processes, elevated UV-B due to ozone depletion has been linked to ecological disruptions and even mass extinction events, such as the end-Permian and Devonian–Carboniferous extinctions (10–13). UV-B is of particular interest because it is thought to reach the Earth’s surface in greater intensity during periods of severe ozone depletion caused by catastrophic events. On a microbial scale, UV radiation has continually shaped the trajectory of evolution over billions of years, from contributing to the origins of life to driving genetic variation and adaptation (14, 15). This is because UV radiation directly damages DNA and induces certain repair mechanisms that are highly mutagenic (16–19).

The UV light spectrum, ranging from 100 to 400 nanometers (nm), can be categorized into four groups: UV-A (315–400 nm), UV-B (280–315 nm), UV-C (100–280 nm), and vacuum-UV (100–200 nm) (20). UV-C is more readily absorbed by nucleic acids compared to UV-B and UV-A (21) and is therefore more detrimental due to the greater extent of DNA damage it induces. When bacteria are exposed to UV radiation (6, 7), it triggers the formation of pyrimidine dimers and other DNA lesions that disrupt the normal structure and function of DNA molecules (22, 23). These UV lesions are removed by various effective bacterial systems, such as the photoreactivation repair system (i.e., dimer monomerization under visible light by photolyase enzymes, such as Phr) (24), base excision repair (BER) (removal of cyclobutane-thymine dimers using DNA glycosylases and AP endonucleases) (25), and the primary pathway for removal of bulky DNA lesions, i.e., the nucleotide excision repair (NER) (for rapid repair of photolesions using the *uvr* genes, *polA*, and ligase enzyme) (26). However, an excessive amount of dimers that cannot be removed by the repair systems can accumulate and interfere with the cellular replication process, resulting in the formation of “secondary” lesions in the form of ssDNA fragments (27). Therefore, to deal with this severe DNA damage, a post-replication damage repair mechanism known as the bacterial SOS response pathway is induced, preventing premature cell division (16, 28, 29) and providing cells with sufficient time to repair the damaged DNA (18, 30–32).

This SOS response in bacteria is controlled by the multi-functional RecA protein, a key component that cleaves the transcriptional repressor LexA (33–35). This cleavage initiates the expression of more than 40 SOS genes. RecA, involved in the recombination repair of ssDNA gaps (36), helps provide an error-free damage removal route. However, excessive DNA damage leads to the induction of the mutagenic phase of the SOS response, mediated by bacterial DNA polymerase enzymes, including Pol V (37, 38), which is encoded by the *umuD* and *umuC* genes (39). Pol V can bypass template lesions during DNA replication through a process known as translesion DNA synthesis (TLS) (40). Two additional TLS DNA polymerases, Pol II (encoded by *polB*) and Pol IV (encoded by *dinB*), facilitate replication past blocking lesions, albeit at the cost of introducing mutations.

Another DNA damage repair strategy is the mismatch repair (MMR) pathway (41), which recognizes base-base mismatches and insertion/deletion loops, including those introduced by DNA polymerases. However, with increased mutagenic stress, the efficiency of MMR decreases, as observed in the case of fluoroquinolone treatment (42). Such stress can potentially activate the SOS response and be addressed by SOS-mediated repair mechanisms, as the accumulation of DNA lesions and stalled replication forks is known to trigger this global DNA damage response.

UV radiation can serve as a valuable experimental tool for investigating mutagenesis because it induces a wide range of mutations without an apparent sequence preference (43, 44). Additionally, UV radiation is of great interest to scientists for its applications in biotechnology, particularly in directed evolutionary strategies to engineer proteins or organisms (45, 46). Moreover, there is growing interest in using UV radiation as a disinfectant, including for sterilizing air, equipment, and surfaces; reducing the transmission of airborne diseases; and even for treating wound infections (47, 48). Despite their increased applications in clinical settings, we believe that two phenomena—UV-induced bacterial cell dormancy and mutagenesis—need to be fully understood. Bacterial cells are highly heterogeneous, and they can enter a growth-arrested state through stochastic mechanisms or environmental factors (49), including UV treatment, which can make these cells highly tolerant. Also, the SOS-response-mediated cell dormancy not only promotes cell survival but also sustains mutagenic processes, as dormant cells may still harbor DNA damage (50, 51). Additionally, UV radiation itself is highly mutagenic; it can accelerate the emergence of more resilient mutants, which could pose a serious threat to public health.

UV-induced repair mechanisms are highly complex as UV can damage many cellular components, including DNA, RNA, lipids, and proteins (52, 53). While the role of the SOS response in mutagenesis has been extensively studied (refer to the reviews in references 16, 30, 32] for more details), our understanding of the downstream repair mechanisms and their contributions to cell survival, culturability, and mutagenesis remains limited. In this study, we focus on UV-B to model environmentally and evolutionarily relevant DNA damage conditions that allow cells to retain viability and enable the investigation of intermediate recovery states, which are difficult to capture under more severe UV-C exposure. Although our recent study showed an almost perfect correlation between RecA levels and mutation frequency in *E. coli* following UV-B treatment, prolonged UV-B exposure was found to impair SOS-mediated mutagenesis and markedly reduce colony-forming unit (CFU) formation on agar medium immediately after treatment (54). However, when these cells were allowed a short recovery period in liquid culture, CFU levels rapidly improved. While this may represent a potential survival strategy for cells to evade UV-B-induced mutagenesis, the underlying mechanisms remain unclear. Our current study aims to investigate these complex mechanisms further, providing new insights into the molecular processes underlying mutagenesis and dormancy.

## RESULTS AND DISCUSSION

### UV-B exposure drives early CFU impairment and dose-dependent mutagenesis in *E. coli*

**Fig 1:**
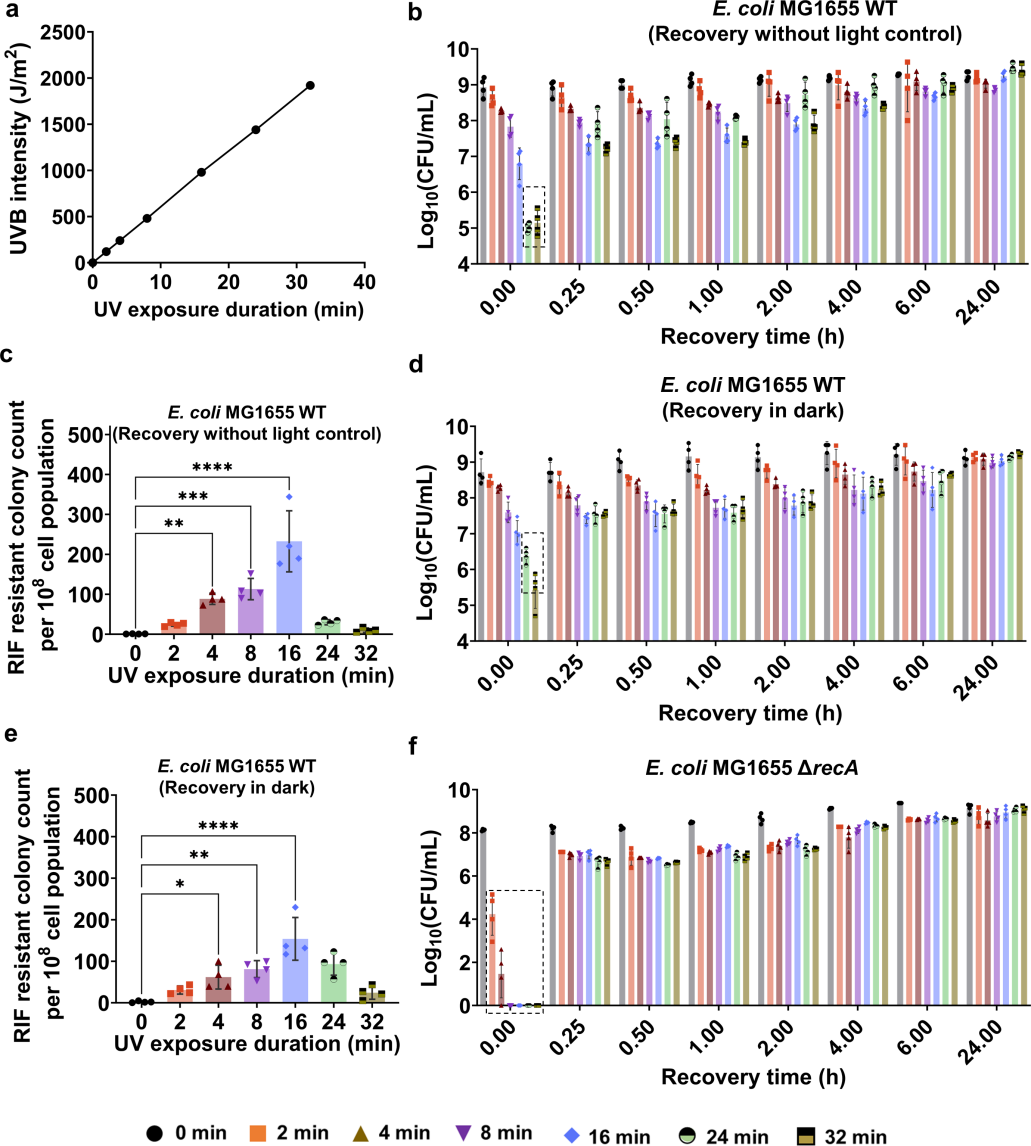
Excessive UV exposure resulted in an early impairment in culturability and decreased mutagenesis. (**a**) The graph shows the relationship between UV-B exposure time (min) and calculated energy dosage (J/m²), based on measured irradiance (see Materials and Methods). (**b**) Exponential-phase *E. coli* MG1655 WT cells were exposed to UV-B light for 0, 2, 4, 8, 16, 24, and 32 min, followed by a 24-hour recovery period. At specific time points during recovery (*t*  =  0 h, 0.25 h, 0.5 h, 1 h, 2 h, 4 h, 6 h, and 24 h), cells were collected and plated to determine colony-forming units (CFU). (**c**) Levels of UV-induced rifampicin (RIF) resistance mutations were measured by counting RIF-resistant colonies (per 10^8^ cells) in the WT cultures after recovery for the indicated UV exposure times. (**d**) CFU counts and (**e**) mutation frequency of *E. coli* MG1655 following UV-B exposure were determined under dark conditions. The same experimental setup described above was used, but cultures were recovered in the absence of light to eliminate photoreactivation. (**f**) The temporal CFU profiles of *E. coli* MG1655 Δ*recA* cells were monitored during recovery following UV treatment. Early CFU impairments are highlighted by boxed regions in panels **b**, **d**, and **f**. *n* = 4. Statistical analysis was performed using one-way ANOVA with Dunnett’s post-test, where *P  <  0.05, **P  <  0.01, ***P  <  0.001, ****P  <  0.0001. Data corresponding to each time point represent mean value  ±  standard deviation.

To determine how UV-B exposure influences mutagenesis and recovery dynamics, we applied our previously established methodology (54) to systematically vary UV-B dose and monitor cellular responses over time. We cultured *E. coli* MG1655 wild-type cells in 2 mL lysogeny broth (LB) medium in test tubes until they reached the mid-exponential phase with an optical density (OD_600_) of ~0.5 (Fig. S1). Subsequently, the cells were transferred to petri dishes, forming a thin film of cultures that increased the surface area for UV exposure. This film was then subjected to UV-B light (302 nm thin-line transilluminator, UVP ChemStudio, Analytik Jena) for various durations: 2, 4, 8, 16, 24, and 32 minutes (min), ensuring a wide range of UV intensity from 120 J/m² to as high as 1920 J/m², as shown in Fig. 1a (see Fig. S2 and Materials and Methods).

Given that UV-B is less energetic than UV-C, we employed a broad range of exposure durations to capture dose-dependent effects while maintaining cell viability for recovery analysis (55–59). If prolonged UV-B exposure primarily impairs colony-forming ability rather than causing immediate cell death, we would expect reduced CFU immediately after treatment, followed by recovery during subsequent incubation. Following exposure, the cells in LB were promptly transferred back to test tubes and cultured in a shaker for a 24-hour recovery (refer to Materials and Methods for details). Cultures that did not receive UV-B treatment served as controls. During recovery, CFU levels are quantified at the indicated time points for each condition, as shown in Fig. 1b.

Consistent with this expectation, immediately after treatment (*t* ~ 0), CFU levels decreased significantly in cultures exposed to longer UV-B treatments (Fig. 1b; Fig. S3a). A 4- to 8-minute exposure caused a 10-fold reduction, while 16 min led to a ~100-fold drop compared to the control. Exposure times of 24 and 32 min resulted in a ~10,000-fold reduction (Fig. 1b). However, this CFU impairment for the 24- and 32-minute UV-B treatments occurred only immediately after exposure; when these treated cells were allowed to recover for ~15 min in liquid medium, CFU levels increased sharply. This drastic increase in CFU levels is not due to cell division, given that the *E. coli* doubling time is about 20–25 min, which is too short for significant growth within that period. By the 24-hour mark, all conditions showed similar CFU levels (Fig. 1b). These results indicate that prolonged UV-B exposure transiently impairs culturability rather than causing irreversible cell death, and that this defect can be rapidly alleviated during recovery.

To determine how UV-B exposure influences mutation accumulation, we quantified mutagenesis by measuring rifampicin (RIF)-resistant colony formation after recovery. Cultures were plated on LB agar with 500 µg/mL RIF, and colony counts were reported per 10^8^ cells (Fig. 1c). We chose a 24-hour recovery period to ensure accurate colony counts, consistent with previous studies (60–62), as mutation levels generally plateau by this time (54). If UV-B induces mutagenesis through DNA damage and error-prone repair, we would expect mutation frequency to increase with UV dose, provided that cells retain the ability to recover and express these mutations. Consistent with this, mutagenesis increased with UV-B exposure, peaking at 16 min. However, longer exposures (24–32 min) significantly reduced RIF-resistant colonies (Fig. 1c). These results suggest that while UV-B promotes mutation formation, prolonged exposure impairs the ability of cells to recover and form colonies, thereby uncoupling mutagenesis from observable resistance.

Although this method does not directly measure all mutations, it is widely used as a proxy for mutagenesis (44, 61, 63, 64) because rifampicin-resistant colony formation strongly correlates with overall mutation frequency (65, 66). To confirm the genetic basis of rifampicin resistance, we performed targeted sequencing of the *rpoB* locus in representative resistant colonies. Consistent with the known rifampicin resistance-determining region (RRDR) (67, 68), mutations were identified within canonical hotspot regions (corresponding to amino acid positions ~507–533 in *E. coli*), including a TCC→TTC substitution resulting in a Ser→Phe amino acid change. In addition, alternative nucleotide substitutions at the same site (e.g., C→A) were observed, indicating that multiple mutational routes can give rise to rifampicin resistance (Table S1).

Our initial experiments were conducted without controlling for light exposure. Given the role of photoreactivation in bacterial recovery, we investigated whether the observed loss and restoration of culturability were driven by photolyase-mediated repair. The *phr* gene encodes DNA photolyase, which reverses UV-induced cyclobutane pyrimidine dimers in the presence of blue light (69, 70). We were unable to obtain viable Δ*phr* knockouts in *E. coli* MG1655 using the λ-Red recombinase technique (see Materials and Methods) (71), which likely reflects strain-specific genetic features of MG1655, as photolyase loss has been reported to be essential in this background (72), whereas it is dispensable in BW25113 (73). We therefore evaluated the role of photoreactivation by repeating the UV-B exposure and recovery experiments under strict dark conditions. Importantly, despite minor fluctuations in mutation frequency—expected due to altered photolyase-mediated repair mechanisms—we observed the same core features under dark conditions: a pronounced decline in culturability, followed by recovery, and a dose-dependent mutation frequency (Fig. 1d and e; Fig. S3b). To directly test whether *phr* function is necessary for these outcomes, we conducted parallel experiments using a Δ*phr* strain from the Keio collection (*E. coli* BW25113 Δ*phr*), as this strain can have a viable knockout *phr* strain. Upon UV-B treatment and recovery, this strain showed a similar reduction in colony formation, along with a dose-dependent mutation frequency (Fig. S4), mirroring the responses seen in MG1655. While some quantitative differences were observed in BW25113 (likely due to the distinct genetic background), these results indicate that the observed phenomena were not solely contingent on light-driven DNA repair. This is plausible, as UV-induced mutations likely arise from error-prone repair mechanisms (such as those mediated by SOS response) rather than error-free photoreactivation by Phr.

### Deletion of *recA* leads to an early impairment in CFU formation after UV-B treatment

In our previous study, we reported that prolonged UV-B exposure (32 min) suppresses the SOS response, including reduced RecA protein levels (54). Building on this, we sought to determine whether the early impairment in CFU formation is associated with changes in SOS activity and cellular morphology. We reasoned that if SOS activation contributes to maintaining culturability, conditions with an active SOS response should exhibit characteristic filamentation and increased cell size, whereas conditions with impaired SOS activity should lack these features. Consistent with this, microscopy analysis in the current study revealed pronounced filamentation after the 16-min UV-B treatment, whereas cells exposed to the 32-min UV-B treatment showed only modest elongation (Fig. S5). These morphological differences aligned with our flow cytometry measurements of forward scatter (FSC), which reflects cell size, and side scatter (SSC), which reflects cell surface complexity and internal structure. Both FSC and SSC increased after the 16-min UV-B treatment, and this increase was not obvious after the 32-min UV-B treatment (Fig. S6). Importantly, the number of singlet events (individual cells gated to exclude aggregates, debris, and dead cells) remained stable across all conditions (Fig. S7), demonstrating that overall cell numbers did not change. Thus, the early decrease and later increase in CFU observed after 32 min of UV-B exposure cannot be explained by filament breakup or the expansion of a minor surviving subpopulation, and instead reflect changes in the ability of cells to form colonies on agar.

The early impairment in CFU formation likely reflects a more complex process involving multiple components of the SOS network, with RecA acting as the central regulator. To test whether this phenotype is driven by impaired SOS response activity, we disrupted the pathway by deleting *recA*, expecting that loss of RecA would further compromise culturability if SOS function is required for recovery. Our results verified that the knockout strain of *recA* exhibited a drastic reduction in culturability immediately after UV-B treatment across all conditions (Fig. 1f; Fig. S3c). Initially, CFU levels in the Δ*recA* strain were below the limit of detection (1 CFU) for most UV treatment conditions; however, this was followed by a significant increase in CFU levels (from 1 to 10^7^) within 15 min of recovery (Fig. 1f; Fig. S3c). This observation is highly interesting, and to our knowledge, such a dramatic recovery pattern in a *recA*-deficient background has not been previously reported. Although liquid-holding recovery has been well documented since the 1960s (74, 75), our findings are distinct because the marked increase in culturability following UV-B treatment occurs in a genetic background lacking a functional SOS response. While our previous work showed that *recA* deletion abolishes mutagenesis across all UV-B exposure durations (54), the mechanisms driving this outcome and its impact on CFU formation remain unclear, representing a key gap addressed in this study.

### Prolonged UV-B exposure impairs transcription, translation, and SOS activation

To identify the mechanisms underlying the observed impairment in the SOS response, we next investigated whether prolonged UV-B exposure disrupts key cellular processes required for DNA damage response. We reasoned that this impairment could arise from growth-arrest pathways, membrane damage, oxidative stress, or inhibition of transcriptional and translational capacity. If these processes are compromised, we would expect reduced gene expression, limited RecA availability, and suppression of SOS activation.

**Fig 2:**
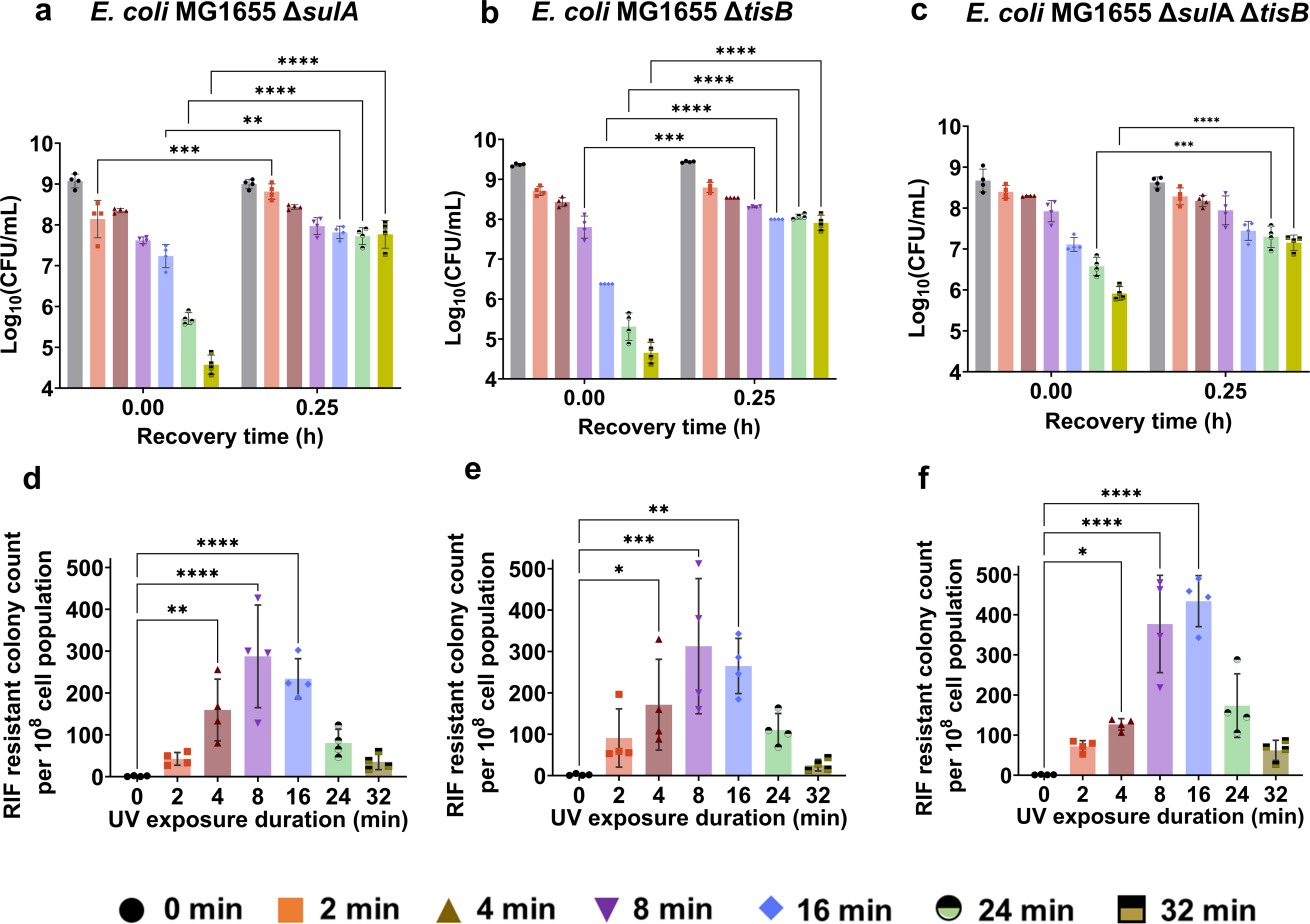
Perturbation of both SulA and TisB proteins moderately increased cell culturability during recovery. (**a–c**) Exponential-phase *E. coli* MG1655 Δ*sulA*, Δ*tisB*, and Δ*sulA*Δ*tisB* cells were exposed to UV-B light for 0, 2, 4, 8, 16, 24, and 32 min, followed by a 24-hour recovery period. At specific time points during recovery, cells were collected and plated to determine their CFU levels. Note that only the 0 and 0.25 h time points are shown here; the full-time course is provided in Fig. S8. (**d–f**) Levels of UV-induced RIF resistance mutations were measured by counting RIF-resistant colonies (per 10^8^ cells) in the cultures of three knockout strains after recovery for the indicated UV exposure times. *n* = 4. Statistical analysis was performed using two-way ANOVA with Šidák’s multiple-comparison test for panels **a–c** and one-way ANOVA with Dunnett’s post-test for panels **d–f**. Significance is indicated as *P  <  0.05, **P  <  0.01, ***P  <  0.001, ****P  <  0.0001. Data corresponding to each time point represents mean value  ±  standard deviation.

We first examined growth-arrest pathways associated with the SOS response. Two key SOS genes, *sulA* and *tisB*, mediate cell growth inhibition: *sulA* encodes a cell division inhibitor that halts the cell cycle to prevent segregation of damaged DNA (76), whereas *tisB* encodes a toxin that reduces ATP levels and can induce reversible dormancy by suppressing cellular metabolism (50, 77). The rationale is that if these growth-arrest pathways contribute to the observed CFU impairment, their deletion would alter recovery dynamics following UV-B exposure. To investigate the individual and combined effects of these genes, we constructed knockout strains of *E. coli* MG1655 Δ*sulA*, Δ*tisB*, and Δ*sulA*Δ*tisB*. These strains, at mid-exponential phase, were exposed to varying durations of UV-B radiation followed by a 24-hour recovery period. *E. coli* MG1655 WT cells were used as the control group. Deleting *sulA* and *tisB* individually did not alter the trend of CFU profiles observed in our experiments. Specifically, CFU levels initially showed a significant decrease after prolonged UV-B exposure of 24 and 32 min, followed by a sharp increase within the first 15 min of recovery, similar to the wild-type strain (Fig. 1b vs Fig. 2a through c). However, CFU levels of Δ*sulA*Δ*tisB* immediately after 24 and 32 min of UV-B exposure (*t* ~ 0) were nearly 10-fold higher compared to those of the wild-type or single knockout strains. We observed approximately a 10-fold increase in CFU levels within the first 15 min of recovery in the double knockout strain, although this increase was less pronounced than in the control or single knockout strains (Fig. 2a through c; Fig. S8). While deleting both genes enhanced the culturability of cells following UV-B treatment, the phenomenon observed in the wild-type strain was not completely eliminated in the double knockout strain, highlighting the complexity of the underlying mechanisms. No distinct profiles of RIF-resistant mutants were observed after 24 h of recovery in either single or double-knockout strains (Fig. 2d through f), showing a broadly dose-dependent response similar to WT, although with some variations. Altogether, these results suggest that these genes may not be directly involved in mutagenesis, while they may influence cellular culturability to some extent.

**Fig 3:**
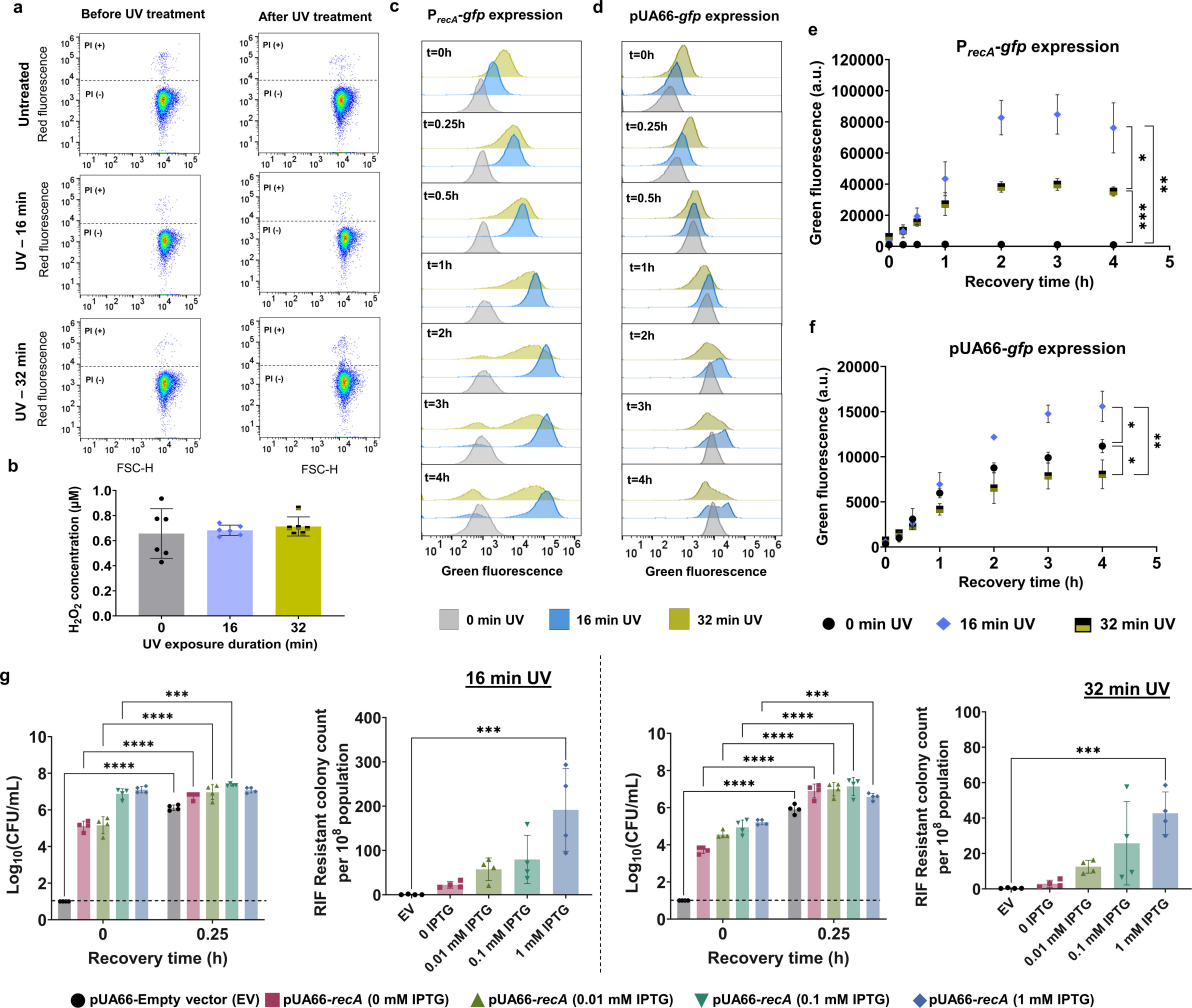
Excessive UV exposure did not compromise cell membrane integrity or increase hydrogen peroxide levels, but it did impair cellular translation. (**a**) Membrane integrity of UV-treated cells (0, 16, and 32 min of UV exposure) was assessed via flow cytometry with PI staining (see Fig. S9 for controls). FSC-H: forward light scatter. (**b**) H_2_O_2_ concentrations were measured using the Amplex Red Hydrogen Peroxide/Peroxidase Assay Kit. Reaction mixtures containing Amplex Red reagent, horseradish peroxidase, and UV-treated cells in sodium phosphate buffer (pH 7.4) were incubated for 30 min at room temperature, followed by fluorescence measurement with a microplate reader. H_2_O_2_ levels were calculated using a standard curve (Fig. S10). (**c**) Expression of P*_recA_-gfp* in UV-treated *E. coli* MG1655 cells was analyzed via flow cytometry for the indicated time points during recovery (P*_recA_*: the *recA* promoter). (**d**) IPTG-inducible GFP expression in UV-treated *E. coli* MG1655 pUA66-*gfp* cells was also assessed by flow cytometry for the indicated time points during recovery. (**e**) GFP expression profiles of UV-treated cells harboring the *recA* promoter and (**f**) IPTG-inducible GFP expression systems were evaluated using mean GFP values from flow cytometry data. (**g**) Recovery (CFU counts) and mutation frequency of *E. coli* MG1655 Δ*recA* carrying an IPTG-inducible *recA* overexpression plasmid were assessed following 16- and 32-minute UV-B exposures, shown for the indicated IPTG concentrations. The dashed lines represent the limit of CFU detection. *n* ≥ 4. Statistical analysis was performed using two-way ANOVA with Greenhouse-Geisser correction and Tukey’s multiple-comparison test for panels **e** and **f**. For panel **g**, recovery data were analyzed by two-way ANOVA with Šidák’s multiple-comparison test, while mutagenesis data were analyzed by one-way ANOVA with Dunnett’s post-test. Significance is indicated as *P  <  0.05, **P  <  0.01, ***P  <  0.001, ****P  <  0.0001. Data corresponding to each time point represent mean value  ±  standard deviation.

We next examined whether prolonged UV-B exposure compromises cellular membrane integrity. UV radiation can damage cellular membranes and disrupt essential cellular functions (19). If membrane damage contributes to the observed CFU impairment, we would expect increased permeability and loss of cellular contents following treatment (78, 79). To test this, cells treated with UV-B were collected at multiple recovery time points (0.25, 0.5, 1, 2, 3, 4, and 24 h) and incubated with propidium iodide (PI), a fluorescent dye that enters cells with compromised membranes. Across all time points examined, flow cytometry revealed no significant increase in PI staining following prolonged UV-B treatment (32 min) compared to untreated cells or moderately UV-treated cells (16 min), both of which did not markedly reduce CFU levels (Fig. 3a presenting representative data from the 1-hour recovery time point; see Fig. S7 for live-cell counts and Fig. S9 for controls).

We then assessed the potential role of oxidative stress in the observed CFU impairment. Prolonged UV exposure can induce the production of reactive oxygen species (ROS), which can damage cellular components and impair cellular function (80–82). The rationale is that if oxidative stress contributes to this phenotype, elevated ROS levels would be detected following prolonged UV-B exposure. When UV-treated samples were subjected to Amplex Red reagent (see Materials and Methods for details) to detect hydrogen peroxide (H_2_O_2_), a primary reactive oxygen species, no significant changes were observed in the 32-minute UV-treated cells compared to untreated or 16-minute UV-treated cells (Fig. 3b; see Fig. S10 for the standard curve), indicating that H_2_O_2_ may not be a key player. In our previous study, we found that ROS significantly affects cell culturability only when their concentrations are much higher than physiological levels, and their impact on cell culturability was observed to be non-transient (54). Although H_2_O_2_ levels did not increase in liquid cultures after prolonged UV-B exposure, this does not exclude the possibility that oxidative stress on solid agar contributes to the early impairment of CFU formation when cells are plated immediately after treatment. To assess this possibility, we supplemented LB agar with increasing amounts of catalase (0, 1,000, 2,000, 4,000, and 8,000 units per plate) and plated cells immediately after the 32-minute UV-B exposure (Fig. S11). If oxidative stress on agar were limiting colony formation, catalase supplementation would be expected to improve CFU levels. However, CFU counts remained unchanged across all catalase conditions and were similar to those on unsupplemented plates (Fig. S11), indicating that oxidative effects on agar are unlikely to account for the early reduction in CFU formation following prolonged UV-B treatment.

Finally, we examined whether prolonged UV-B exposure impairs transcriptional and translational capacity. UV can block RNA polymerase progression by inducing pyrimidine dimers and other photoproducts in DNA (83) and can also damage ribosomal RNAs through UV-driven crosslinking (84), potentially inhibiting gene expression. If these processes are compromised, reduced transcription and translation would limit RecA availability and suppress SOS response. To assess this, we analyzed GFP expression under the control of the *recA* promoter (P*_recA_*) at the single-cell level using *E. coli* MG1655 cells carrying the pUA66 P*_recA_-gfp* plasmid. The cells were subjected to UV-B treatments for 16 min and 32 min, and *recA* expression was measured using flow cytometry during the early recovery phase (Fig. 3c). UV-B treatment should activate promoters of SOS response genes and induce *recA* expression. As expected, the 16-minute UV-B treatment resulted in significantly higher *recA* expression compared to untreated cells (Fig. 3c and e). However, the 32-minute UV-B exposure resulted in more heterogeneous and lower *recA* expression compared to the 16-minute UV-B exposure (Fig. 3c and e), potentially implying an impairment in the transcription and translation processes. This difference between the 16- and 32-minute treatments cannot be solely attributed to increased cell death following 32 min of UV-B exposure. Despite the early impairment in CFU formation in the prolonged UV-exposure culture, CFU levels in both conditions are nearly similar after a brief recovery period of about 15 min (see the 0.25-hour time point in Fig. 1b). To substantiate the hypothesis regarding the impairment of transcription and translation processes, we employed a GFP-expressing plasmid where the GFP gene is tightly regulated by an isopropyl β-D-1-thiogalactopyranoside (IPTG)-inducible T5 promoter and a strong LacI^q^ repressor (85). Our rationale was that if excessive UV-B treatment indeed impairs these processes, the levels of GFP expression in cells treated with 32 min of UV-B should be lower compared to those in the no-UV control or the 16-minute UV-treated cells, despite the presence of the inducer. We supplemented cell cultures with 0.1 mM IPTG immediately before UV-B treatments. As anticipated, the 32-minute UV-B treatment resulted in a lower level of GFP expression compared to the no-UV control or the 16-minute UV-B treatment (Fig. 3d and f). Of note, the differences in GFP output between the two reporters (P*_recA_-gfp* vs T5-*gfp*; Fig. 3c and d) are expected because the T5 promoter is synthetic and drives strong IPTG-induced expression, whereas P*_recA_* is a native SOS promoter that responds to physiological regulation. P*_recA_-gfp* is more sensitive to UV-B–induced disruptions in transcription and translation, while the T5-gfp system serves as an independent high-expression control confirming that excessive UV-B treatment can still impair these processes.

To further validate the role of the RecA-mediated SOS response in recovery and mutagenesis following UV-B exposure, particularly under conditions where transcription and translation are impaired, we constructed an IPTG-inducible *recA* expression system. This was achieved by cloning *recA* under the control of a strong T5 promoter (Fig. S12) and introducing the plasmid into the *E. coli* MG1655 Δ*recA* background. As expected, in the absence of induction, the strain behaved like a *recA* knockout, showing impaired recovery and loss of UV-induced mutagenesis (Fig. 3g). However, we also noticed that cells carrying the plasmid displayed improved culturability even without IPTG induction. Based on our previous work, the T5 promoter is known to be strong and slightly leaky (86), and this basal expression likely provided sufficient RecA to partially restore culturability, even after 32 min of UV-B treatment. To further probe this effect, we added IPTG approximately 30 min before UV exposure to ensure that RecA protein was already present, thereby avoiding possible interference from UV-induced transcription/translation defects. Increasing IPTG concentrations led to a graded restoration of both CFU levels and mutation frequency following 16- and 32-minute UV-B exposures (Fig. 3g). The improvement in culturability was evident even at lower IPTG levels, while mutagenesis showed a particularly clear and dose-dependent increase with higher induction.

Altogether, our findings suggest that while prolonged UV-B treatments do not markedly affect membrane integrity or ROS levels, the early impairment of CFU formation likely arises from the combined effects of cellular stress responses, including growth-arrest pathways, reduced translational capacity, and disruptions in downstream repair processes. Notably, our data indicate that prolonged UV-B exposure reduces the SOS response, as reflected by lower *recA* expression (Fig. 3c). This interpretation is strongly supported by our genetic analyses, which show that loss of *recA* causes pronounced defects in both CFU formation and UV-induced mutagenesis, while restoring RecA activity through overexpression improves both processes (Fig. 3g).

### Genes significantly expressed during the SOS response have a moderate or negligible effect on cell culturability and mutagenesis

Although our results indicate that RecA availability is a key determinant, the specific contributions of downstream SOS genes remain less defined. If these genes play functional roles in the observed phenotypes, their expression profiles should correlate with recovery and mutagenesis outcomes. To examine this, we screened a subset of the *E. coli* promoter library, in which native promoters are fused to a fast-folding GFP reporter on low-copy plasmids, enabling quantitative measurement of gene expression (87). We focused on approximately 40 promoters that regulate key SOS and SOS-associated genes under RecA-LexA control, selected based on a comprehensive analysis of *E. coli* regulatory databases (73). In these experiments, *E. coli* MG1655 cells carrying individual promoter-GFP fusions were grown to mid-exponential phase in 96-well plates and then exposed to 16 min of UV-B. We chose 16-minute exposure as it induces strong SOS activation and high mutagenesis without severely affecting culturability (Fig. 1b and c), making it an appropriate condition for probing promoter activity. GFP levels were measured after 24 h of recovery. Although SOS promoter induction is short-lived, the GFP used in this reporter system is stable, allowing UV-induced promoter activity to accumulate to detectable levels over time (Fig. S13). Because many SOS reporters exhibit relatively low activity and are difficult to quantify with a plate reader, we used 24-hour, high-cell-density cultures to ensure reliable fluorescence measurements in this high-throughput screening format (Fig. S13).

**Fig 4:**
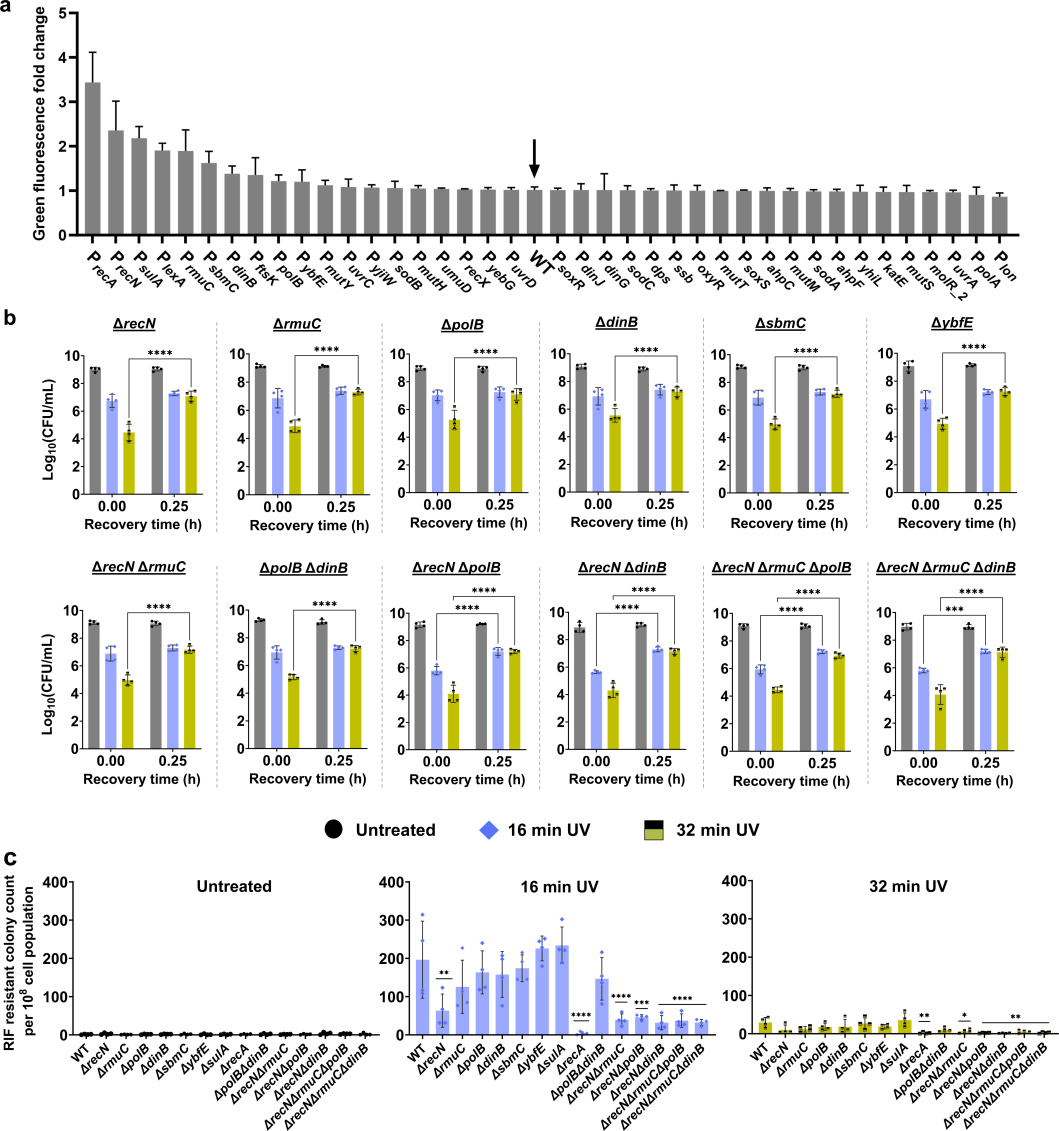
UV-induced upregulation of SOS genes and its impact on mutagenesis and cell culturability. (**a**) A library of *E. coli* MG1655 strains with promoter reporters for SOS response genes was UV-treated during the mid-exponential phase and allowed to recover for 24 h. GFP levels were measured after recovery and normalized to untreated controls. WT cells without any promoter reporters were used as background controls. (**b**) Genes upregulated following UV exposure were individually or combinatorially deleted in *E. coli* MG1655, and a mutagenesis assay was performed. Mid-exponential phase cells were exposed to UV for 0, 16, and 32 min and recovered for 24 h. At specified time points during recovery (*t*  =  0 h, 0.25 h, 0.5 h, 1 h, 2 h, 4 h, 6 h, and 24 h), cells were collected and plated to determine CFU. Note that only the 0 and 0.25 h time points are shown here; the full-time course is provided in Fig. S14. (**c**) RIF-resistant cells were quantified by plating samples on RIF-agar plates after 24 h of recovery, with results reported as RIF-resistant colony counts per 10^8^ cells for different UV exposure durations. *N* = 4. Statistical analysis was performed using two-way ANOVA with Šidák’s multiple-comparison test for panel **b** and one-way ANOVA with Dunnett’s post-test for panel **c**, where each knockout strain was compared directly to the wild type (WT). Significance is indicated as *P  <  0.05, **P  <  0.01, ***P  <  0.001, ****P  <  0.0001. Data corresponding to each time point represent mean value  ±  standard deviation.

Our specific focus was on genes showing an upregulation following UV treatment (Fig. 4a), as they likely play pivotal roles in the downstream mechanisms of RecA-mediated SOS response. Several promoters showed moderate upregulation, including P*_recA_* and P*_lexA_* (promoters of genes encoding SOS response global regulators, as expected), P*_recN_* and P*_rmuC_* (promoters of genes involved in DNA recombinational repair), P*_sulA_* (promoter of a gene encoding cell division inhibitor), P*_polB_* and P*_dinB_* (promoters of genes encoding DNA polymerase enzymes), P*_ftsK_* (promoter of a gene encoding essential cell division protein), P*_sbmC_* (promoters of a gene encoding inhibitor of DNA gyrase-mediated DNA supercoiling), and P*_ybfE_* (promoter of a *lexA-*regulated gene whose function is not well characterized)(88) (Fig. 4a). To determine whether the genes associated with the identified promoters are involved in SOS response-mediated culturability and mutagenesis, we generated single deletions in *E. coli* MG1655 for these genes (except for *lexA* and *ftsK*, as they are essential and could not be deleted) (89–91). The knockout strains were exposed to UV-B for 16 min or 32 min or untreated, and their CFU profiles during recovery, as well as their RIF-resistant colony levels, were similarly measured. For all knockout strains, the temporal CFU profiles remained the same as that of the wild type, showing an early impairment in CFU formation when cells were plated immediately after the 32-minute UV-B exposure, with CFU levels improving after 15 min of recovery (Fig. 4b; Fig. S14). As expected, the deletion of *recA* completely eliminated mutagenesis (Fig. 4c). Although 32 min of UV-B exposure significantly reduced mutagenesis in all strains, including wild type, the two DNA recombination gene knockouts, Δ*recN* and Δ*rmuC*, showed a 3-fold and 1.5-fold decrease in RIF-resistant mutant levels, respectively, compared to WT cells following 16 min of UV-B treatment (Fig. 4c). Surprisingly, other single deletion strains, including those for the genes encoding DNA polymerase II and IV, *dinB* and *polB*, did not show significant changes (Fig. 4c).

Overall, our results showed that single deletions of the downstream genes of RecA did not drastically impact cellular culturability and mutagenesis, except for RecN and RmuC deletions, which slightly reduced mutagenesis. RecN is involved in the recombinational repair of DNA double-strand breaks, and its mutation makes cells sensitive to mitomycin C and ionizing radiation (92–94). Although RmuC is thought to be an inner membrane protein with a nuclease domain (95, 96), its function is not well understood, and our data indicate it only moderately impacts UV-B-mediated mutagenesis. It is quite surprising that the deletion of DNA polymerase II and IV, two key proteins involved in indirect mutagenesis (97), did not impact UV-B-induced mutagenesis. Deleting a single polymerase gene may have minimal impact because the remaining polymerase can functionally compensate for its loss. Given that mutagenesis is a multi-faceted process controlled by multiple mechanisms, testing multi-deletion strains may be necessary to reveal their combined effects. Therefore, we first generated Δ*recN*Δ*rmuC* and showed that the double deletion resulted in a cumulative decrease (~5-fold) in RIF-resistant colony levels compared to the wild type following 16 min of UV-B treatment (Fig. 4c). To assess the impact of DNA polymerase II and IV, we generated a Δ*polB*Δ*dinB* mutant strain, which did not affect RIF-resistant colony levels (Fig. 4c). Additionally, we generated Δ*recN*Δ*polB* and Δ*recN*Δ*dinB* double knockout strains and demonstrated that they exhibited approximately a 5-fold reduction in RIF-resistant colony levels compared to wild-type cells (Fig. 4c). Deletion of DNA polymerase genes *polB* and *dinB* from Δ*recN*Δ*rmuC* individually did not show significant changes compared to the Δ*recN*Δ*rmuC* strain in RIF-resistant mutant levels (Fig. 4c), suggesting that the observed reduction in RIF-resistant colonies in multi-deletion strains is primarily due to the effects of *recN* and/or *rmuC*.

### Screening the knockout strains reveals the redundancy of repair mechanisms in UV-induced mutagenesis and culturability

**Fig 5:**
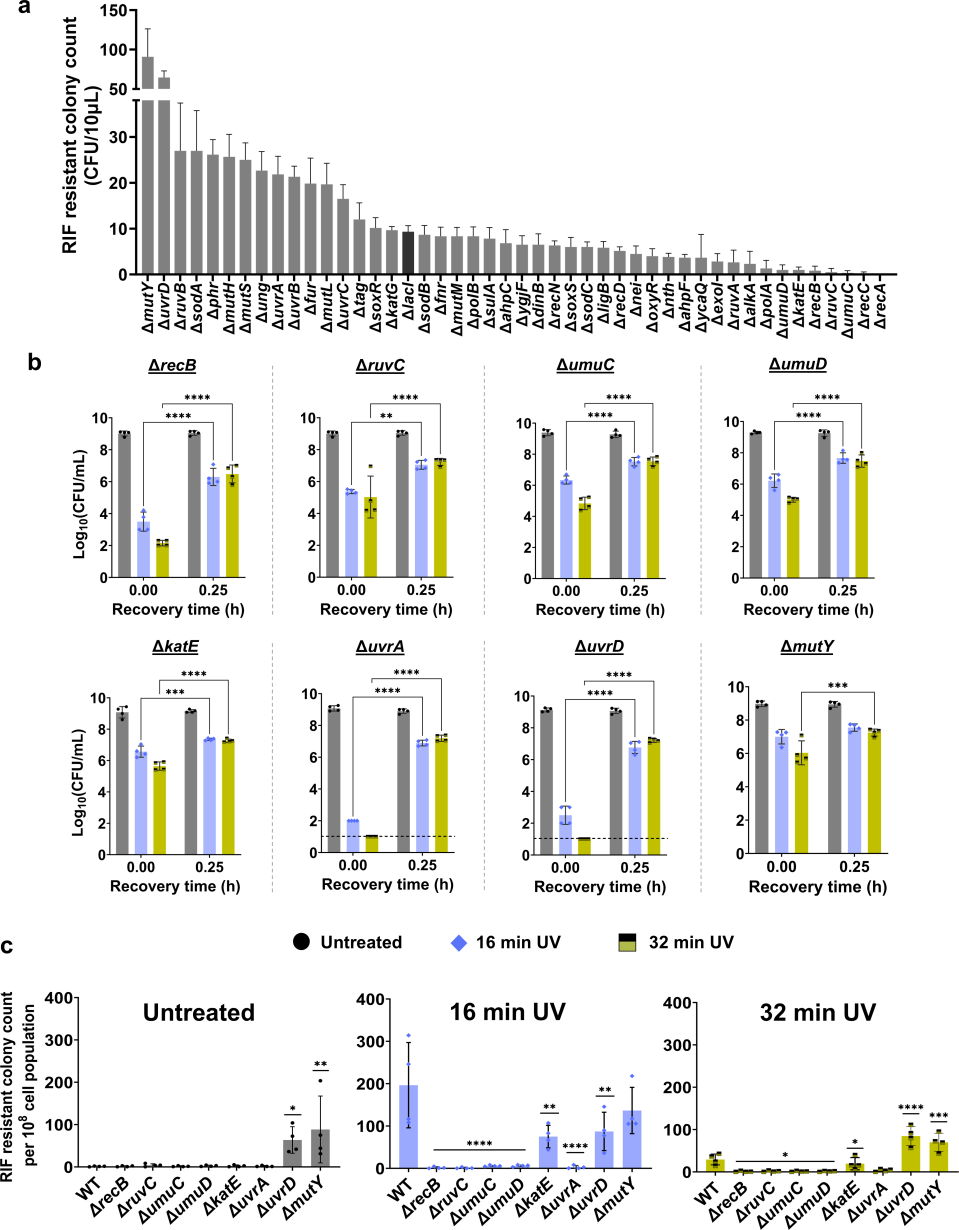
Knockout library screening to elucidate their effects on mutagenesis and cell culturability. (**a**) The *E. coli* BW25113 Keio knockout collection was screened following 16 min of UV treatment. RIF-resistant cells were quantified by spotting 10 μL samples from each well onto RIF-agar plates after a 24-hour recovery period. Δ*lacI* was used as the reference control (see Materials and Methods). (**b**) Selected genes were deleted in *E. coli* MG1655, and the mutagenesis assay was performed. Mid-exponential phase cells were exposed to UV for 0, 16, and 32 min and recovered for 24 h. At indicated time points (*t* =  0 h, 0.25 h, 0.5 h, 1 h, 2 h, 4 h, 6 h, and 24 h) during recovery, cells were collected and plated to enumerate CFU. Note that only the 0 and 0.25 h time points are shown here; the full-time course is provided in Fig. S15. The dashed lines represent the limit of CFU detection. (**c**) RIF-resistant cells were quantified by plating the samples on RIF-agar plates after 24 h of recovery. The values were reported as RIF-resistant colony count per 10^8^ cell population for different UV exposure durations. *n* = 4. Statistical analysis was performed using two-way ANOVA with Šidák’s multiple-comparison test for panel **b** and one-way ANOVA with Dunnett’s post-test for panel **c**, where each knockout strain was compared directly to the wild type (WT). Significance is indicated as *P  <  0.05, **P  <  0.01, ***P  <  0.001, ****P  <  0.0001. Data corresponding to each time point represents mean value  ±  standard deviation.

To obtain a more comprehensive understanding of the repair mechanisms underlying the observed phenotype, we conducted a second screening using the *E. coli* BW25113 Keio knockout collection. This approach enabled us to systematically assess the functional contributions of a broader set of DNA repair pathways, including (i) those not regulated by RecA (e.g., mismatch repair) and (ii) RecA-regulated genes that may not have appeared strongly upregulated in the initial screen due to the limited sensitivity of plate reader-based measurements. If these pathways contribute to mutagenesis and culturability, their deletion would be expected to alter mutation frequency and/or recovery following UV-B exposure (Fig. 4a). For this second screening, the corresponding Keio knockout strains in 96-well plates were treated with UV-B for 16 min during mid-exponential-phase growth, and RIF-resistant colonies were quantified after a 24-hour recovery period. We identified several genes whose deletion resulted in a significant reduction in RIF-resistant colony formation (Fig. 5a), including *recB*, a component of exonuclease V or the RecBCD complex, which promotes homologous recombination in the repair of double-strand DNA breaks (98, 99); *umuC* and *umuD*, encoding error-prone DNA polymerase V (100); *ruvC*, encoding an endonuclease that binds to and cleaves Holliday junctions (101); and *katE*, encoding *E. coli* catalase enzyme (102). Additionally, we observed several genes exhibiting increased mutagenesis, such as *uvrD* and *uvrA*, which are involved in the nucleotide excision repair pathway (103, 104), and *mutY*, involved in the base excision repair pathway (105) (Fig. 5a). Knockouts of MMR genes (*mutS*, *mutL*, *mutH*, and *exoI*), which function in replication error correction, were included in our screen but did not show significant reductions in mutagenesis, indicating MMR may play a minimal role under our UV-B exposure conditions (Fig. 5a).

To further investigate genes that showed the most pronounced impact on UV-induced mutagenesis in our screening and to determine if similar trends occur in other *E. coli* strains, we deleted these genes in *E. coli* MG1655 and analyzed the knockout strains after 16 and 32 min of UV-B exposure. Deletion of *recB*, *ruvC*, *umuC*, and *umuD* significantly reduced mutagenesis (Fig. 5c), consistent with our screening data. The absence of UmuC and UmuD proteins, specialized for translesion synthesis, was shown to sensitize *E. coli* cells to UV (106, 107), aligning with our findings. While Δ*uvrA* in the *E. coli* BW25113 strain from screening showed higher mutant levels compared to wild-type cells, its deletion in the MG1655 background resulted in a significant reduction in mutant formation (Fig. 5c), suggesting that the role of certain genes in mutagenesis can vary depending on the genetic background of the *E. coli* strain. Also, our results indicate that KatE, UvrD, and MutY may not be directly involved in the mutagenic response to UV radiation under the conditions tested here, as their deletions did not clearly impact UV-B-induced mutagenesis in *E. coli* MG1655 (Fig. 5c). Interestingly, Δ*uvrD* and Δ*mutY* strains exhibited increased RIF-resistant colonies in cultures not treated with UV-B (Fig. 5c). UvrD is involved in various DNA repair pathways, including NER and MMR processes (41, 108, 109). MutY is a glycosylase enzyme that corrects adenine mismatches resulting from DNA replication errors, primarily via the BER pathway (105). While the specific roles of UvrD and MutY in UV-B-induced mutagenesis are not evident in this study, the increased number of RIF-resistant colonies in untreated cultures might highlight their potential role in maintaining genomic stability under normal growth conditions, possibly by mitigating spontaneous mutations.

The temporal CFU profiles of Δ*ruvC*, Δ*umuC*, and Δ*umuD* strains were similar to the wild type (Fig. 5b; Fig. S15). However, Δ*recB* exhibited a notable decrease in CFU levels, while Δ*uvrA* and Δ*uvrD* mutants showed even more drastic reductions following UV-B treatment compared to the wild type (Fig. 5b). This reduction occurred when cells were plated immediately after treatment, and their levels increased approximately 10^7^-fold within 15 min of recovery, similar to Δ*recA* profiles. UvrA and UvrD together form key components of the NER pathway (108). UvrA forms a complex with UvrB to detect DNA lesions, while UvrD functions as a helicase to unwind and remove damaged DNA segments (109, 110). As both UvrA and UvrD are regulated by the RecA protein (32), it is not surprising that the deletion of *recA* resulted in drastic reductions in CFU levels following UV treatment (Fig. 1f). Since RecA is essential for inducing the SOS response, its absence means that many DNA repair genes, including *uvrA* and *uvrD*, are not upregulated in response to UV-induced DNA damage. This was further validated when we measured the expression levels of *uvrD* and *uvrA*, which were reduced under 32 min of UV treatment compared to 16 min (Fig. S16a through d), consistent with the inhibition of transcription/translation and reduced RecA expression under extended UV exposure (Fig. 3c and e). Notably, *uvrA* and *uvrD* expression levels are not very high overall (Fig. S16a through d), which may explain why these differences were not detected in our plate reader–based screen. Altogether, the immediate reduction in CFU levels in the knockout strains (Δ*recA*, Δ*uvrA*, and Δ*uvrD*), as well as in the wild-type strain exposed to prolonged UV, highlights the importance of the SOS response in the initial phases of DNA damage repair. The similar outcomes in all these mutants also suggest that *E. coli* possesses redundant or numerous repair mechanisms (16) that can partially restore culturability even when certain DNA repair pathways are disrupted.

### Conclusions

Our findings reveal several important aspects of how *E. coli* responds to UV-B stress. First, we show that prolonged UV-B exposure leads to a sharp reduction in CFU upon immediate plating, yet CFU levels recover after short incubation in liquid culture, highlighting an early impairment in colony-forming ability that is alleviated during the recovery period. This conclusion is supported by time-resolved recovery assays and flow cytometry showing stable cell numbers across treatments, thereby excluding filament breakup or survivor expansion as alternative explanations. Second, we identify impaired transcription/translation as a critical driver of this phenomenon, given that reduced expression capacity limits RecA availability, suppresses SOS activation, and uncouples mutagenesis from culturability. Although *recA* and *umuCD* have long been implicated in UV-C mutagenesis, our study shows an important outcome by linking RecA dosage and transcriptional/translational capacity to survival outcomes under UV-B stress. Finally, by systematically analyzing genetic knockouts, we distinguish the differential contributions of DNA repair pathways, showing that genes such as *recB*, *umuC*, *umuD*, and *ruvC* are critical for mutagenesis, whereas *uvrA* and *uvrD* are important for maintaining culturability. Together, these results advance a mechanistic framework in which UV-B stress regulates mutagenesis and culturability through distinct but interconnected pathways.

Beyond these mechanistic insights, our findings have broader implications for understanding how environmental stress shapes microbial evolution and adaptation. The dose-dependent balance between mutagenesis and survival suggests that intermediate stress levels may maximize evolvability by promoting genetic diversity while preserving viability, whereas excessive stress can suppress adaptive potential by disrupting core cellular processes. This has direct relevance to the emergence of resistance, where sublethal stresses may facilitate the generation and selection of resistant mutants, whereas more severe conditions may limit their formation despite extensive damage. More generally, our results highlight how global physiological constraints, such as transcriptional and translational capacity, can act as key regulators of evolutionary outcomes, extending beyond classical DNA repair pathways. These principles may also inform strategies in biotechnology and directed evolution, where controlled stress conditions are used to optimize mutation rates while maintaining population viability.

### Future directions

Our findings indicate that prolonged UV-B exposure triggers a reversible loss of colony-forming ability that becomes more pronounced when the SOS response is impaired, as observed in the *recA* deletion strain. While this provides a direct link between DNA damage responses and early impairment in CFU formation, cells appear to employ distinct survival strategies in liquid versus solid media. Growth on agar may activate additional pathways (beyond oxidative stress, which was not sufficient to explain the reduced culturability under our conditions), and these pathways may operate redundantly and in conjunction with SOS-mediated responses. Determining the specific mechanisms that govern survival and recovery on agar, particularly when the SOS response is compromised, will require extensive and systematic genetic and physiological analyses that focus exclusively on agar-grown cells. It may also be informative to conduct comparative studies between liquid and solid media to identify pathways that are uniquely activated in each condition. Single-cell analyses could further reveal how heterogeneous responses contribute to both survival and mutagenesis in liquid versus agar environments. These questions lie outside the scope of the present study and warrant investigation in a dedicated, independent effort.

## MATERIALS AND METHODS

### Bacterial strains and plasmids

*Escherichia coli* K-12 MG1655 wild type (WT), the pUA66 plasmid containing an IPTG-inducible *gfp* (green fluorescent protein gene) expression system, and the pUA66 plasmid with *gfp* under the control of the *recA* promoter (P*_recA_*) were obtained from Dr. Mark P. Brynildsen at Princeton University. *E. coli* K-12 MG1655 Δ*sulA*, Δ*tisB*, Δ*recA* was constructed in our previous studies (54, 111, 112). The promoter strain collection of *E. coli* MG1655, in a 96-well plate format used for the screening assay, was obtained from Horizon Discovery (Lafayette, CO, USA). The Keio knockout strain collection (a single-gene deletion library of *E. coli* K-12 BW25113) was obtained from Dharmacon Keio Collection (Dharmacon, catalog no. OEC4988). Since the knockout strains carry a kanamycin resistance gene, high-throughput screening was performed in the presence of kanamycin to prevent contamination; thus, Δ*lacI* was used as the reference control, given that the parental BW25113 strain lacks kanamycin resistance. We selected the Δ*lacI* mutant because it is not involved in DNA repair pathways and serves as a neutral background. Furthermore, its response to UV-B exposure closely mirrors that of the MG1655 wild-type strain (Fig. S4a), supporting its suitability as a functional reference for our experimental comparisons. The strains from both the promoter and knockout collections used in this study are listed in Table S2. The knockout *E. coli* MG1655 strains generated for this study are detailed in Table S3. The method of Datsenko and Wanner (71) was used to generate these strains, and the oligonucleotides used to delete the genes are provided in Table S4. We also attempted to generate a Δ*phr* mutant in *E. coli* MG1655 using the same approach; however, no viable recombinants were obtained despite testing multiple primer sets with varying homology arm lengths (40–60 bp) (Table S4). An IPTG-inducible *recA* overexpression plasmid was generated using a commercial cloning service (Synbio Technologies, USA). The *recA* coding sequence from *E. coli* MG1655 was cloned downstream of a T5 promoter into a low-copy plasmid backbone (pUA66) containing a strong, mutated *lacI* repressor for tight regulation and a kanamycin resistance marker (112). The resulting plasmid was verified by sequencing and subsequently transformed into MG1655 Δ*recA* cells for inducible expression studies. The plasmid map is shown in Fig. S12.

### Chemicals, media, and culture conditions

Unless otherwise specified, all chemicals were procured from Fisher Scientific (Atlanta, GA), VWR International (Pittsburgh, PA), or Sigma Aldrich (St. Louis, MO). PI staining kit was purchased from Promega Corporation (Madison, WI). *E. coli* cells were cultured in liquid Lysogeny-Broth (LB) medium. LB agar medium was utilized for enumerating colony-forming units (CFU) of *E. coli*. The liquid LB medium was prepared by dissolving 5 g yeast extract, 10 g tryptone, and 10 g sodium chloride in 1 L of deionized (DI) water. LB agar media were prepared by dissolving 40 g of pre-mixed LB agar in 1 L of DI water. Both solid and liquid media were subjected to autoclaving for sterilization.

Kanamycin (50 µg/mL) was included in the liquid LB medium for plasmid selection and retention. IPTG at 0.1 mM was used to induce *gfp* expression. For PI staining, sterile 0.85% sodium chloride solution was used. When necessary, cells were washed with phosphate-buffered saline (PBS; 1×). Stock solutions for rifampicin (RIF; 50 mg/mL) were prepared by dissolving in DI water using 0.01 N sodium hydroxide. The IPTG stock solution (1 mM) was dissolved in DI water. All chemical solutions were sterilized using 0.2 μm VWR syringe filters.

To prepare RIF-agar plates, the stock RIF solution was added to autoclaved LB agar, resulting in a final plate concentration of 500 μg/mL RIF. Unless specified otherwise, overnight pre-cultures were generated in 14 mL Falcon test tubes containing 2 mL of liquid media. These pre-cultures were inoculated from 25% glycerol cell stocks stored at −80°C and cultivated for 24 h at 37°C with shaking at 250 revolutions per minute (rpm). Experimental cell cultures were prepared by diluting the overnight pre-cultures (1:100) into 2 mL of fresh LB medium in 14 mL Falcon test tubes. Bacterial cells in this study reached the mid-exponential phase (OD_600_ ~0.5) after around 3 h, attaining an average cell density of 7 × 10^8^ CFUs/mL. All treatments involving UV were administered at this stage.

### UV treatment and cell recovery

Overnight pre-cultures of *E. coli* MG1655 cells were diluted 100-fold in 2 mL fresh LB medium in test tubes and grown at 37°C with shaking (250 rpm). Cell growth was monitored by measuring optical density at 600 nm (OD_600_) using a plate reader (Varioskan LUX Multimode Microplate Reader, Thermo Fisher, Waltham, MA, USA). When the cell density reached OD_600_ ~0.5, cultures from the test tubes were transferred to petri dishes (the diameter of petri dishes = 100 mm, catalog no. FB0875713, Fisher Scientific). This procedure created a thin film of culture in the dish with a height of about 0.25 mm. This configuration resulted in a uniformly distributed liquid film without observable surface tension-driven droplet formation (i.e., beading). The cultures were exposed to a UV-B light source (302 nm; UVP ChemStudio, catalog no. 849-97-0928-02; Analytik Jena, Jena, Germany) for varying exposure times (0, 2, 4, 8, 16, 24, and 32 min). In this setup, UV-B light was emitted from below using a transilluminator and passed through a UV-permeable plastic petri dish placed directly on the transilluminator surface. This configuration ensured uniform and efficient bottom-up exposure of bacterial cells within the thin culture film. To verify the consistency and intensity of UV-B irradiation, direct irradiance measurements were performed using a Vernier UV-B Sensor (Fisher Scientific, Cat. No. S16273ND), calibrated for peak sensitivity at 315 nm (detection range: 290–320 nm), in combination with a LabQuest 3 data acquisition platform. Measurements were taken under four conditions to assess the impact of the experimental setup on UV transmission: (i) sensor placed directly on the transilluminator surface without a petri dish, (ii) sensor on an empty petri dish, (iii) sensor inside a petri dish containing LB medium (not in contact with the liquid), and (iv) sensor in direct contact with the LB medium inside the dish. In all cases, UV-B irradiance was consistently measured at approximately 1,000 mW/m² (equivalent to 1 W/m²), indicating that neither the petri dish nor the medium significantly interfered with UV transmission (see Fig. S2). The energy dose (in J/m²) for each exposure condition was calculated using the following formula: energy dose (J/m²) = irradiance (W/m²) × exposure time (s).

After UV exposure for the indicated time points, cells with the LB medium were transferred back to test tubes and recovered for 24 h. During the recovery period, 10 μL samples were collected at specified time points (*t* = 0, 0.25, 0.5, 1, 2, 3, 4, 6, and 24 h) from each test tube, serially diluted in PBS in round-bottom 96-well plates, and then plated on LB agar media. Whenever necessary, particularly for samples showing a significant reduction in culturability, 100 μL or 1,000 μL of culture was plated immediately after treatment (*t* ~ 0) to increase the limit of detection. The plates were incubated at 37°C for 16 h to enumerate CFU. We note that new colonies were not formed when incubated beyond 16 h. A similar procedure was followed for the other strains. For dark recovery experiments, all procedures prior to UV treatment were identical to those described above. Following UV exposure, samples were incubated in a large, dark incubator-shaker, with the front window fully covered in aluminum foil to prevent light exposure and photoreactivation. Additionally, the test tubes containing the cultured cells were completely wrapped in aluminum foil to ensure total darkness during the recovery period.

### Assessing mutagenesis

To assess the extent of mutant cell formation induced by UV exposure, cells were exposed to UV radiation for the specified durations, followed by a 24-hour recovery period. After this recovery period, 500 μL of cells were collected and spread onto agar plates containing 500 μg/mL RIF. These plates were then incubated at 37°C for 16 h to enumerate RIF-resistant colonies. To determine clonogenic survival, CFU levels were determined, and mutant levels were normalized by dividing the number of RIF-resistant colonies by the total number of colonies in a 1 mL culture. Unless specified otherwise, mutant formation was reported as the count of RIF-resistant colonies per 10^8^ cell population.

### Targeted sequencing of *rpoB* locus

To identify mutations associated with rifampicin resistance, targeted sequencing of the *rpoB* locus was performed. Mid-exponential phase *E. coli* MG1655 wild-type cultures were subjected to UV-B treatment (16 min) following the protocol described above, while untreated cultures were used as controls. After a 24-hour recovery period, cells were plated on LB agar plates containing 500 μg/mL rifampicin. Individual resistant colonies were isolated, and four independent colonies from four biological replicates were selected for analysis.

Genomic regions spanning approximately 1,000–2,000 nucleotides of the *rpoB* gene were amplified by PCR. This region was selected to encompass the rifampicin resistance-determining region (RRDR), which contains well-characterized mutational hotspots (e.g., corresponding to amino acid positions ~507–533 in *E. coli*) where substitutions are known to disrupt rifampicin binding to RNA polymerase and confer resistance (67, 68). Including a broader flanking region ensured coverage of both canonical hotspots and nearby sites that may also contribute to resistance. PCR products were verified by agarose gel electrophoresis and purified using gel extraction prior to sequencing.

Amplicon sequencing was performed using the Plasmidsaurus amplicon sequencing service (South San Francisco, CA, USA) with the premium PCR and cleanup option. This service utilizes Oxford Nanopore-based long-read sequencing technology to generate high-coverage consensus sequences from PCR amplicons. For premium PCR samples, library preparation was performed using a ligation-based (native barcoding) approach, which preserves full-length DNA molecules and enables sequencing of individual amplicons without fragmentation. Sequencing was carried out on a Nanopore platform, in which single DNA molecules pass through protein nanopores and are base-called from changes in ionic current signals. Raw sequencing reads were processed by the service provider using internal basecalling and consensus-generation pipelines to produce polished consensus sequences. Typically, several thousand reads per sample are obtained, resulting in consensus sequences with high accuracy (>99.99%).

Consensus sequences were aligned to the *E. coli* MG1655 reference *rpoB* sequence using the NCBI BLAST (Basic Local Alignment Search Tool) platform to identify nucleotide substitutions in the amplified region.

### Microscopy analysis

Mid-exponential phase *E. coli* MG1655 cells were exposed to UV-B for the specified durations and allowed to recover in liquid media, as described above. At the indicated time points, 100 µL samples were collected from the recovery cultures, applied to PBS agarose pads, and imaged immediately. PBS agarose pads were prepared as described previously (112). Following sample application, cells were spread evenly across the pad and air-dried near a flame for ~15 min. Glass coverslips (25 × 75 × 0.17 mm) were then gently placed on top of the pads and secured at the corners with tape to maintain stability during imaging. Phase-contrast images were captured using an EVOS FL Auto 2 fluorescence microscope (Thermo Fisher Scientific; catalog no. AMAFD2000) equipped with a 100× oil-immersion objective (Olympus; catalog no. AMEP4733; working distance, 0.3 mm).

### Flow cytometry measurements and PI staining

Mid-exponential *E. coli* cells were treated with UV for 16 min and 32 min, following the protocol described above. At multiple recovery time points (*t* = 0, 0.25, 0.5, 1, 2, 3, and 4 h), UV-treated cells in LB medium were diluted 20-fold in 1.0 mL 0.85% NaCl solution in flow cytometry tubes (5 mL round-bottom Falcon tubes, size: 12  ×  75 mm) to achieve the desired cell density (~10^6^–10^7^ cells/mL) for flow cytometry analysis. The resulting cell suspensions were treated with 20 µM PI dye. PI produces red fluorescence upon binding DNA; however, it can only penetrate cells with damaged membranes. The samples were incubated in the dark at 37°C for 15 min before analysis using a conventional bench-top flow cytometer (NovoCyte 3000RYB, ACEA Biosciences Inc., San Diego, CA, USA). For flow cytometry analysis, we used a slow sample flow rate (14 µL/min) to achieve a sample stream diameter (i.e., core diameter) of 7.7 µm. The instrument has a constant sheath flow rate of 6.5 mL/min. The flow cytometer utilizes low-power solid-state lasers. Cells were excited at a 561 nm wavelength, and red fluorescence was detected using a 615/20 nm bandpass filter. At least 30,000 events were recorded for each sample. NovoExpress software was used to collect the data. PI-stained dead cells, obtained after ethanol (70% [vol/vol]) treatment, were used as a positive control. PI-stained live cells were used as a negative control. Forward and side scatter signals of untreated live cells were used to determine the cells on flow cytometry diagrams, while the positive and negative controls were used to gate PI-positive (+) and PI-negative (−) cell populations (Fig. S9).

### H_2_O_2_ measurement

The Amplex Red Hydrogen Peroxide/Peroxidase Assay Kit (Invitrogen, Thermo Fisher Scientific, catalog no. A22188) was used to assess the amount of H_2_O_2_ produced in the UV-treated cells. The Amplex Red Hydrogen Peroxide/Peroxidase Assay Kit contains a sensitive, one-step assay that uses the Amplex Red reagent (10-acetyl-3,7-dihydroxyphenoxazine) in combination with horseradish peroxidase (HRP) to detect hydrogen peroxide (H_2_O_2_). In the presence of peroxidase, the Amplex Red reagent reacts with H_2_O_2_ in a 1:1 stoichiometry to produce the red-fluorescent oxidation product, resorufin. Resorufin has excitation and emission maxima of approximately 571 nm and 585 nm. For preparing the stock solutions, 10 mM Amplex Red reagent was prepared by dissolving the contents of the vial of Amplex Red reagent in 60 μL of DMSO. Reaction buffer was diluted 5-fold to prepare 1× reaction buffer, and 10 U/mL horseradish peroxidase (HRP) was prepared by dissolving one vial of HRP in 1.0 mL of 1× reaction buffer. The working solution of 100 μM Amplex Red reagent and 0.2 U/mL HRP was prepared by adding 50 μL of 10 mM Amplex Red reagent stock solution and 100 μL of 10 U/mL HRP stock solution to 4.85 mL of 1× reaction buffer. The assay volume for this experiment was 100 μL. Mid-exponential *E. coli* cells were treated with UV for 16 min and 32 min. Immediately after treatment, 50 μL of sample for each condition was serially diluted in 1× reaction buffer to determine the optimal amount of sample for the assay. Fifty microliters of the Amplex Red reagent/HRP working solution was added to each microplate well containing the samples. The samples were incubated at room temperature for 30 min and protected from light. At the end, the fluorescence was measured using a microplate reader equipped for excitation in the range of 530–560 nm and fluorescence emission detection at ~590 nm. For the standard curve, a 20 mM hydrogen peroxide (H_2_O_2_) working solution was prepared by diluting 3.0% (0.88 M) H_2_O_2_ in 1× reaction buffer, and then further diluting 20 mM H_2_O_2_ working solution into 1× reaction buffer to produce H_2_O_2_ concentrations of 0 to 10 μM, each in a volume of 50 μL. Subsequently, 50 μL of the Amplex Red reagent/HRP working solution was added to each microplate well containing the standards, followed by 30 min of incubation, after which fluorescence was measured using the same method as for the samples (Fig. S1,0).

To assess whether oxidative stress on solid medium contributes to reduced CFU formation immediately following prolonged UV-B exposure, LB agar plates were supplemented with catalase prior to plating. Catalase (≥20,000 U/mL stock) was prepared by dissolving the enzyme in deionized water, followed by filter sterilization. Aliquots of the catalase stock solution were spread evenly onto the surface of LB agar plates and allowed to air-dry to achieve final doses of 1,000, 2,000, 4,000, and 8,000 units per plate, respectively. Control plates did not have any catalase (no supplementation). Mid-exponential phase *E. coli* MG1655 cultures were exposed to 32 min of UV-B as described above. Following treatment, 10 µL samples were serially diluted in 90 µL PBS, and 10 µL of each dilution was immediately plated onto catalase-supplemented and control LB agar plates. This dilution scheme allowed clear colonies to be observed in the first and second dilution spots on the agar (Fig. S11). Plates were incubated under standard conditions, and CFUs were enumerated to assess recovery.

### Fluorescent protein expression assay for reporter genes

Overnight pre-cultures of *E. coli* MG1655 cells carrying reporter genes fused to the SOS gene promoters (P*_recA_*, P*_uvrA_*, and P*_uvrD_*) were diluted 1:100 in 2 mL of LB medium within test tubes and incubated at 37°C with shaking (250 rpm). Upon reaching the mid-exponential phase, the cells were subjected to UV radiation for specified durations (16 min and 32 min). An untreated culture was used as a control. At the beginning of the recovery, 20 µM PI dye was added to the cultures for continuous monitoring of the membrane permeability. At specified time points during the recovery (*t* = 0, 0.25, 0.5, 1, 2, 3, and 4 h), UV-treated cells were diluted 20-fold in 1.0 mL 0.85% NaCl solution in flow cytometry tubes (5 mL round-bottom Falcon tubes, size: 12  ×  75 mm) to achieve the desired cell density (~10^6^–10^7^ cells/mL). The samples were analyzed using the same flow cytometry method described above (see “Flow cytometry measurements and PI staining” above); however, cells were analyzed using two lasers. For measuring the red fluorescence from PI dye, cells were excited at a 561 nm wavelength, and red fluorescence was detected using a 615/20 nm bandpass filter. For measuring the green fluorescence, cells were excited at a 488 nm wavelength, and the green fluorescence was detected using a 530/30 bandpass filter.

### Transcription/translation activities of pUA66-*gfp* plasmid

To assess the effect of the UV treatment on transcription and translation, the amount of GFP produced by *E. coli* strains from the low-copy plasmid was measured. The plasmid, pUA66-*gfp*, has a *gfp* gene under the control of a strong, IPTG-inducible T5 promoter and a strong LacI^q^ repressor. Overnight pre-cultures of *E. coli* MG1655 cells carrying pUA66-*gfp* were diluted 100-fold in 2 mL fresh LB medium in test tubes and grown at 37°C with shaking (250 rpm). At the mid-exponential phase (OD_600_ ~0.5), 0.1 mM IPTG was added to the test tubes, which were then immediately exposed to UV treatment for 16 min and 32 min. Untreated cultures receiving IPTG only (without UV exposure) served as controls. At specified time points during the recovery (*t* = 0, 0.25, 0.5, 1, 2, 3, and 4 h), UV-treated cells were diluted 20-fold in 1.0 mL PBS in flow cytometry tubes (5 mL round-bottom Falcon tubes, size: 12  ×  75  mm) to achieve the desired cell density (~10^6^–10^7^ cells/mL). The samples were analyzed using the same flow cytometry method described above (see “Flow cytometry measurements and PI staining” above); however, cells were analyzed with a laser emitting light at 488 nm, and green fluorescence was detected using a 530/30 bandpass filter.

### Promoter library screening

Overnight pre-cultures were prepared by inoculating the strains from the promoter library into the wells of 96-well plates containing 200 μL LB medium and 50 µg/mL kanamycin (for plasmid retention). The plates were sealed with a sterile, oxygen-permeable membrane (Breathe-Easier, catalog no. BERM-2000, VWR International) and cultured for 24 h at 37°C with shaking at 250 rpm. Overnight pre-cultures were diluted 1:40 in LB medium with kanamycin in a new 96-well plate, sealed, and incubated at 37°C with shaking at 250 rpm. At the mid-exponential phase (OD_600_ = 0.5), cultures in 96-well plates were exposed to UV light (UVP ChemStudio, catalog no. 849-97-0928-02; Analytik Jena, Jena, Germany) for 16 min and then recovered for 24 h at 37°C with shaking at 250 rpm. GFP was measured using a Varioskan LUX Multimode Microplate Reader (Thermo Fisher, Waltham, MA, USA) at the indicated times, with untreated cultures as a control. The excitation and emission wavelengths for GFP measurement were 485 nm and 511 nm, respectively. Fold changes for GFP (treated/untreated cultures after 24-hour treatment) were used to report the promoter activity. The top 10 promoters showing the highest expression were selected for subsequent experiments.

### Screening *E. coli* BW25113 Keio knockout collection

Overnight cultures of single mutants (Table S2) carrying kanamycin resistance genes were diluted 40-fold into flat-bottom 96-well plates, with each well containing 200 μL of cell culture. Kanamycin (50 µg/mL) was added to overnight and treatment cultures to prevent contamination. The plates were securely sealed using a sterile, oxygen-permeable membrane (Breathe-Easier, catalog no. BERM-2000, VWR International) and incubated at 37°C with agitation at 250 rpm. When the cultures reached the mid-exponential phase (OD_600_ ~0.5), the cells in the 96-well plates were exposed to UV light (UVP ChemStudio, catalog no. 849-97-0928-02; Analytik Jena, Jena, Germany) for 16 min. Subsequently, they were allowed to recover for 24 h at 37°C with shaking at 250 rpm. To quantify UV-induced mutant cells, 10 μL samples were collected from each well and spotted onto LB agar plates containing 500 μg/mL RIF at the end of the 24-hour recovery period. The plates were then incubated at 37°C for 16 h to enumerate RIF-resistant colonies. The mutant formation was reported as the count of RIF-resistant colonies per 10 μL of cell culture.

### Overexpression of *recA* using an IPTG-inducible plasmid

Overnight pre-cultures of *E. coli* MG1655 Δ*recA* cells harboring the pUA66-*recA* plasmid were diluted 1:100 into 2 mL of fresh LB medium in test tubes and incubated at 37°C with shaking at 250 rpm. After 2.5 h of growth, IPTG was added to final concentrations of 0.01, 0.1, and 1 mM. One tube was maintained without IPTG as a non-induced control. Cultures were then incubated for an additional 30 min to allow *recA* expression prior to UV exposure. Subsequently, the cultures were exposed to UV for either 16 or 32 min. As a control, *E. coli* MG1655 Δ*recA* cells carrying the pUA66 empty vector were included for both UV exposure durations. During the recovery period, 10 μL samples were collected at defined time points from each culture, serially diluted in PBS using round-bottom 96-well plates, and plated on LB agar to determine CFU. Plates were incubated at 37°C for 16 h. RIF-resistant colonies were enumerated following the same procedure.

### Statistical analysis and reproducibility

For all pairwise comparisons against a control, one-way ANOVA with Dunnett’s post-test was used. For time-course and multi-factor analyses, either two-way ANOVA with Šidák’s multiple-comparison test or repeated-measures (RM) two-way ANOVA with Greenhouse–Geisser correction and Tukey’s multiple-comparison test were applied, as specified in the individual figure legends. A minimum of four independent biological replicates (unless otherwise specified) were conducted for experiments involving UV exposure. In all figures, data corresponding to each time point represent the mean value ± standard deviation. Regarding statistical significance analysis, the threshold values were set as follows: *P  <  0.05, **P  <  0.01, ***P  <  0.001, and ****P  <  0.0001. All figures were created using GraphPad Prism 10.0.2, and the statistical analyses were carried out using GraphPad Prism 10.0.2 statistical functions. FlowJo V 10.7.1 was used to analyze the data obtained from flow cytometry.

## Data Availability

All data presented in this paper are available in the main text or the Supplemental File. All raw data have been published on Figshare: https://doi.org/10.6084/m9.figshare.27701493.v4.

## SUPPLEMENTAL MATERIAL

Supplemental File msystems.00396-26-s0001.pdf Supplemental figures and tables.

ASM does not own the copyrights to Supplemental Material that may be linked to, or accessed through, an article. The authors have granted ASM a non-exclusive, world-wide license to publish the Supplemental Material files. Please contact the corresponding author directly for reuse.
