# Supplementary material for: UV-B-induced DNA repair mechanisms and their effects on mutagenesis and culturability in Escherichia coli
Source: mSystems. 2026 Jun 12;11(7):e00396-26. doi: 10.1128/msystems.00396-26 (PMC13386880; doi:10.1128/msystems.00396-26)
Supplement: Supplemental File — Supplemental figures and tables. [file msystems.00396-26-s0001.pdf]

Supplementary Information

UV-Induced DNA Repair Mechanisms and Their Effects on Mutagenesis and Culturability in *Escherichia coli*

Sreyashi Ghosh[1], Jenet Narzary[1,2], and Mehmet A. Orman[1,2,*]

[1]Department of Chemical and Biomolecular Engineering, University of Houston, United States
[2]Department of Biomedical Engineering, University of Wisconsin-Madison, United States
*Corresponding author: maorman@wisc.edu

Supplementary figures

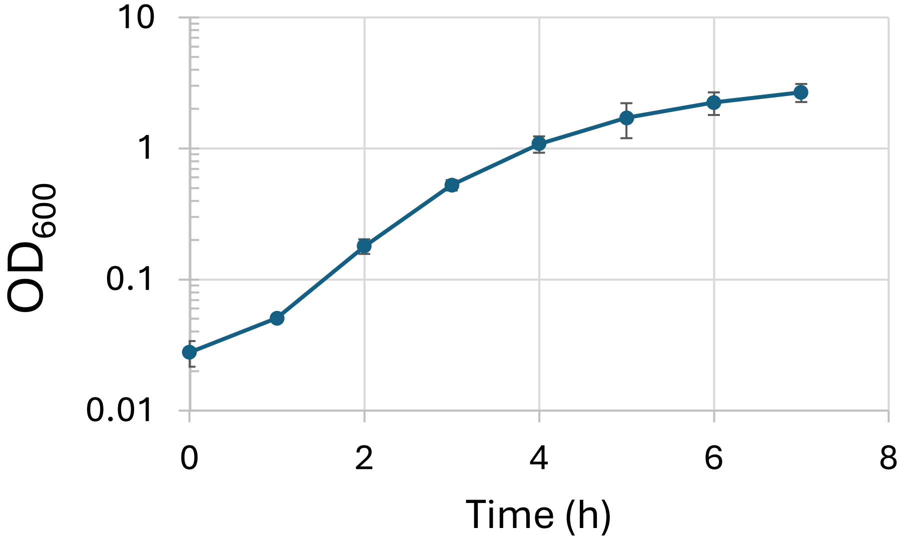

Fig. S1: Cell growth of *E. coli* MG1655. Overnight cultures were diluted into fresh medium and grown under the conditions described in Materials and Methods. Optical density at 600 nm (OD_{600}) was measured at the indicated time points using a plate reader. n=3. Data corresponding to each time point represent mean value ± standard deviation.

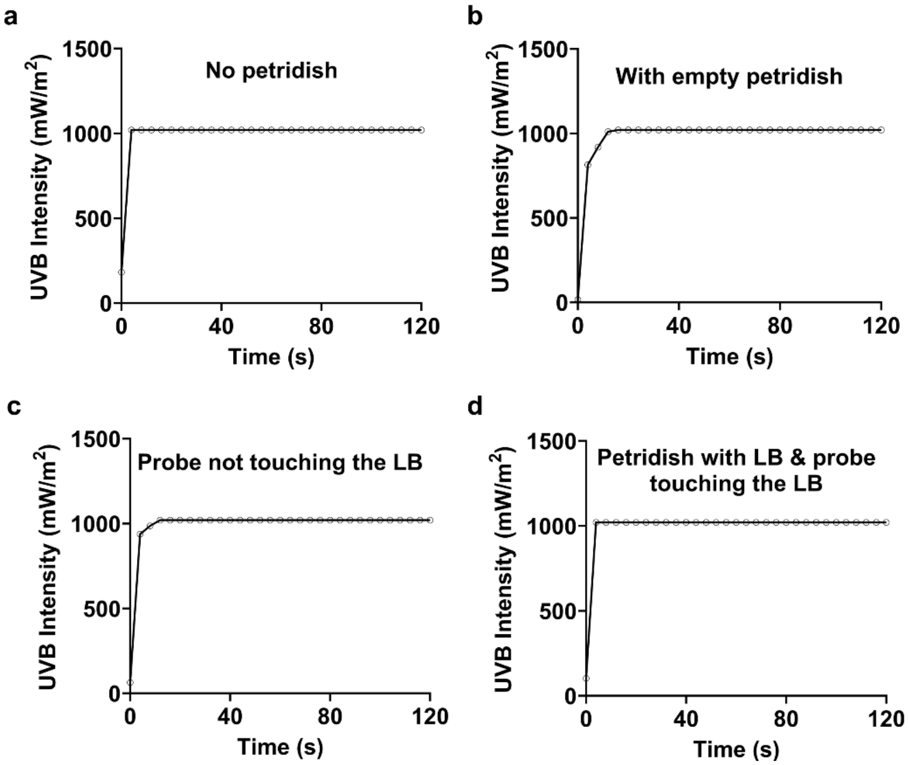

Fig. S2: UV lamp intensity measurement at 302 nm using a UV-B dosimeter. UV-B intensity was measured using a dosimeter (see Materials and Methods for specifications) under four different conditions to ensure accurate assessment of the irradiance reaching bacterial cells. (a) Probe placed directly on the surface of the UV transilluminator block with no Petri dish. (b) Probe placed on an empty Petri dish positioned on the UV transilluminator. (c) Probe positioned inside a Petri dish containing LB medium, without contacting the liquid surface. (d) Probe in direct contact with the LB medium inside the Petri dish on the UV transilluminator block.

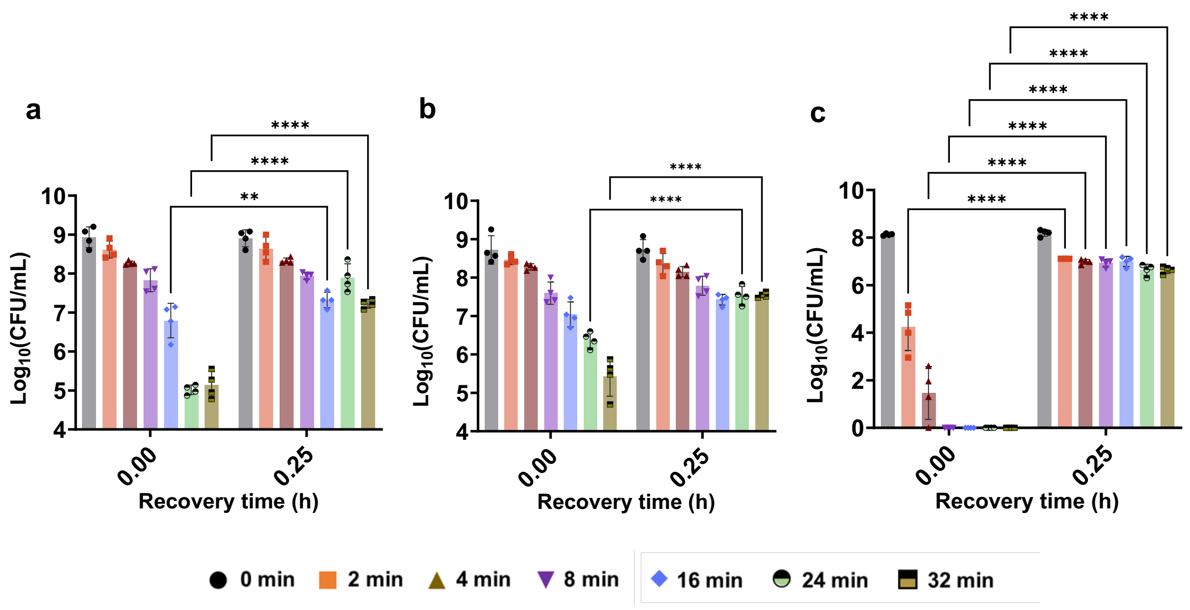

Fig. S3: Statistical analysis of early recovery (0–0.25 h) corresponding to Fig. 1b, d, and f. Log_{10}(CFU/mL) values at early recovery time points (0 and 0.25 h) following UV-B exposure for the indicated treatment conditions. Data correspond to the same biological replicates shown in Fig. 1b, d, and f. Recovery data were analyzed using two-way ANOVA with Šidák's multiple-comparison test, comparing 0 and 0.25 h within each treatment condition. Data are presented as mean ± SD from independent biological replicates. Statistical significance is indicated as *$P < 0.05$, **$P < 0.01$, ***$P < 0.001$, and ****$P < 0.0001$.

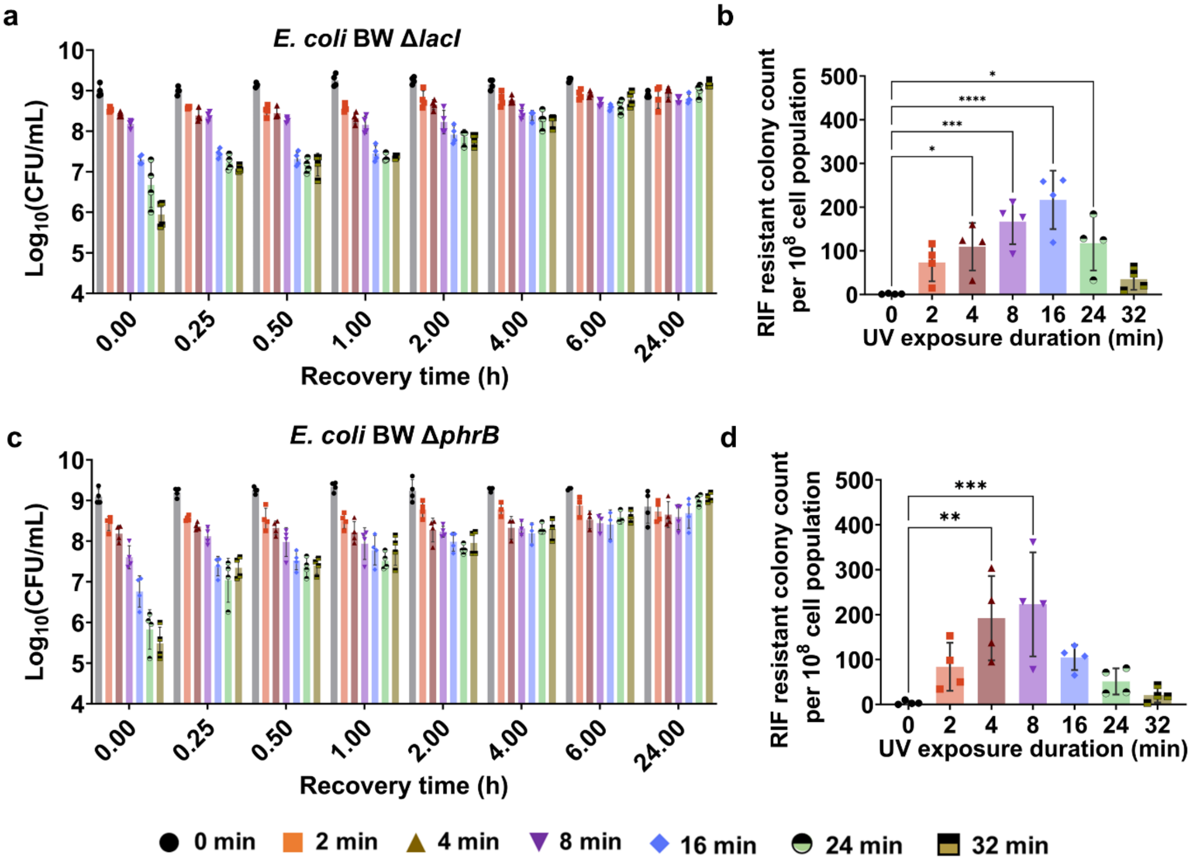

Fig. S4: Photoreactivation-deficient strain *E. coli* K-12 BW25113 Δ*phrB* exhibited similar trends in survival and mutagenesis compared to the Δ*lacI* control strain. (a, c) Exponential-phase *E. coli* BW25113 Δ*lacI* and Δ*phrB* cells were exposed to UV-B radiation for 0, 2, 4, 8, 16, 24, and 32 minutes, followed by a 24-hour recovery period. At designated time points, cells were collected and plated to determine CFU levels. (b, d) UV-induced mutagenesis was assessed by quantifying RIF-resistant colonies (per 10^8 cells) in each knockout strain after recovery at the indicated UV exposure times. Δ*lacI* was used as a reference control. n=4. Statistical analysis was performed using one-way ANOVA with Dunnett's post-test, where *$P < 0.05$, **$P < 0.001$, ***$P < 0.01$, ****$P < 0.0001$. Data corresponding to each time point represent mean value ± standard deviation.

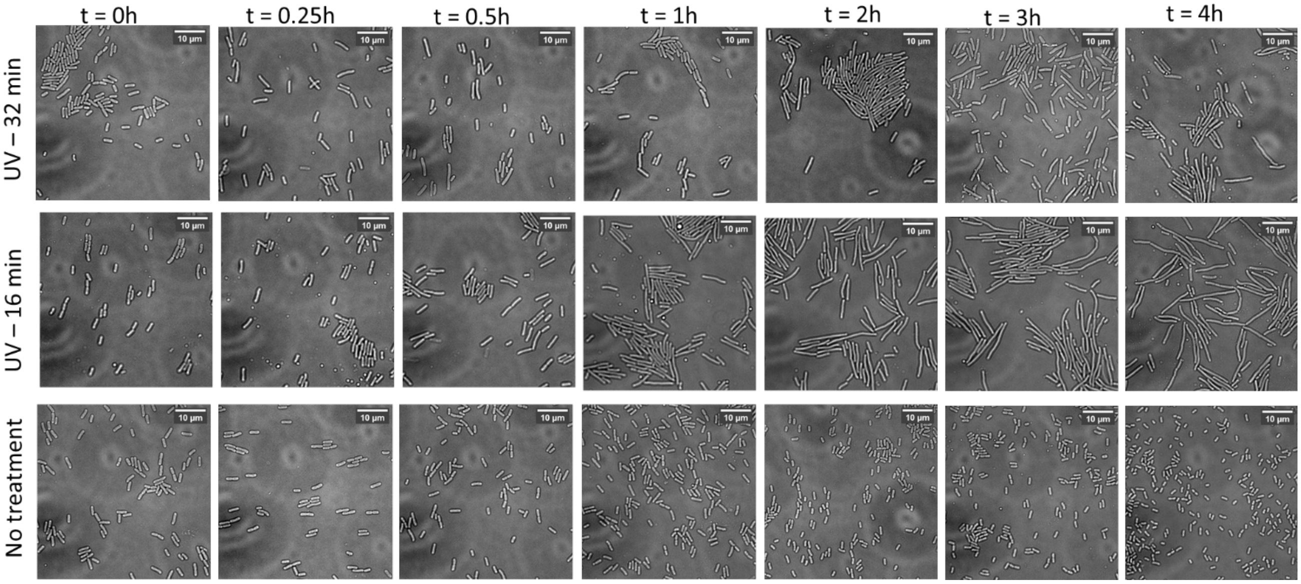

Fig. S5. Microscopy analysis of *E. coli* cell morphology following UV-B treatment. Mid-exponential phase *E. coli* MG1655 cells were exposed to 0 min (no-treatment control), 16 min (UV-16 min), or 32 min (UV-32 min) of UV-B and allowed to recover in liquid media. Samples were collected at the indicated time points, applied to agarose pads, and bright-field microscopy images were acquired immediately after exposure. Scale bar, 10 µm. Cells treated for 16 min exhibited pronounced filamentation, consistent with SOS activation and impaired cell division, whereas cells treated for 32 min displayed only modest elongation.

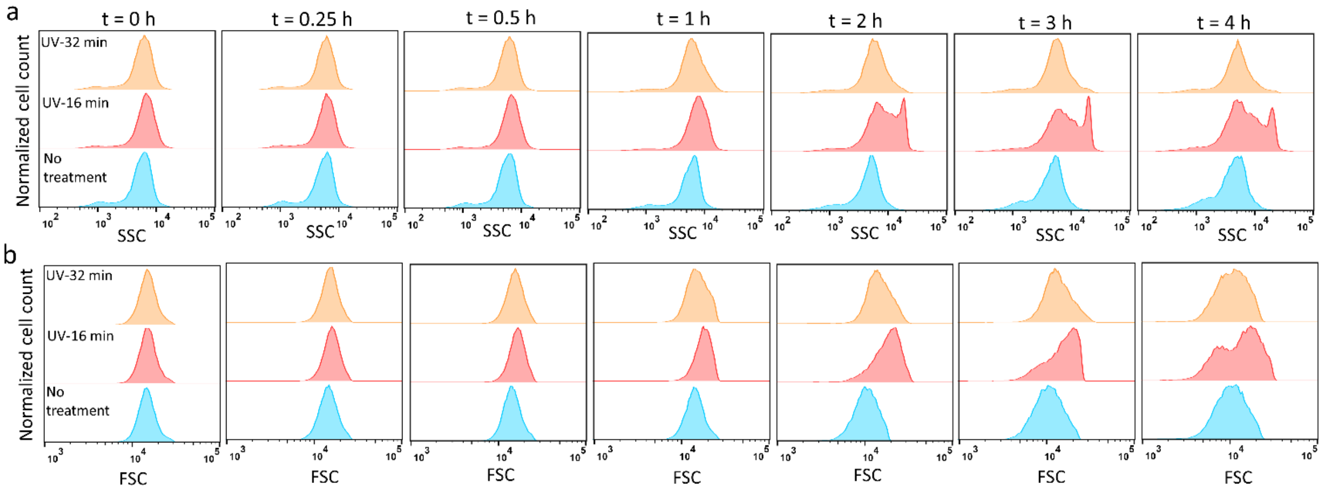

Fig. S6: Forward scatter (FSC) and side scatter (SSC) profiles of *E. coli* cells following UV-B treatment. (a) Flow cytometry SSC distributions measured at 0, 0.25, 0.5, 1, 2, 3, and 4 h of recovery for untreated control, 16-min UV-B-treated, and 32-min UV-B-treated cells. (b) Flow cytometry FSC distributions measured at the same recovery time points for untreated control, 16-min UV-B-treated, and 32-min UV-B-treated cells.

Note: Given the limited dynamic range of FSC for rod-shaped bacteria and its sensitivity to particle orientation, structural alterations associated with early SOS activation are most apparent in 16-min UV-B-treated cells, relative to both untreated cells and 32-min UV-B-treated cells. While microscopy offers higher spatial resolution (see Fig. S5), flow cytometry provides population-level measurements and still captures the pronounced heterogeneity and cell elongation observed in 16-min UV-B-treated cells.

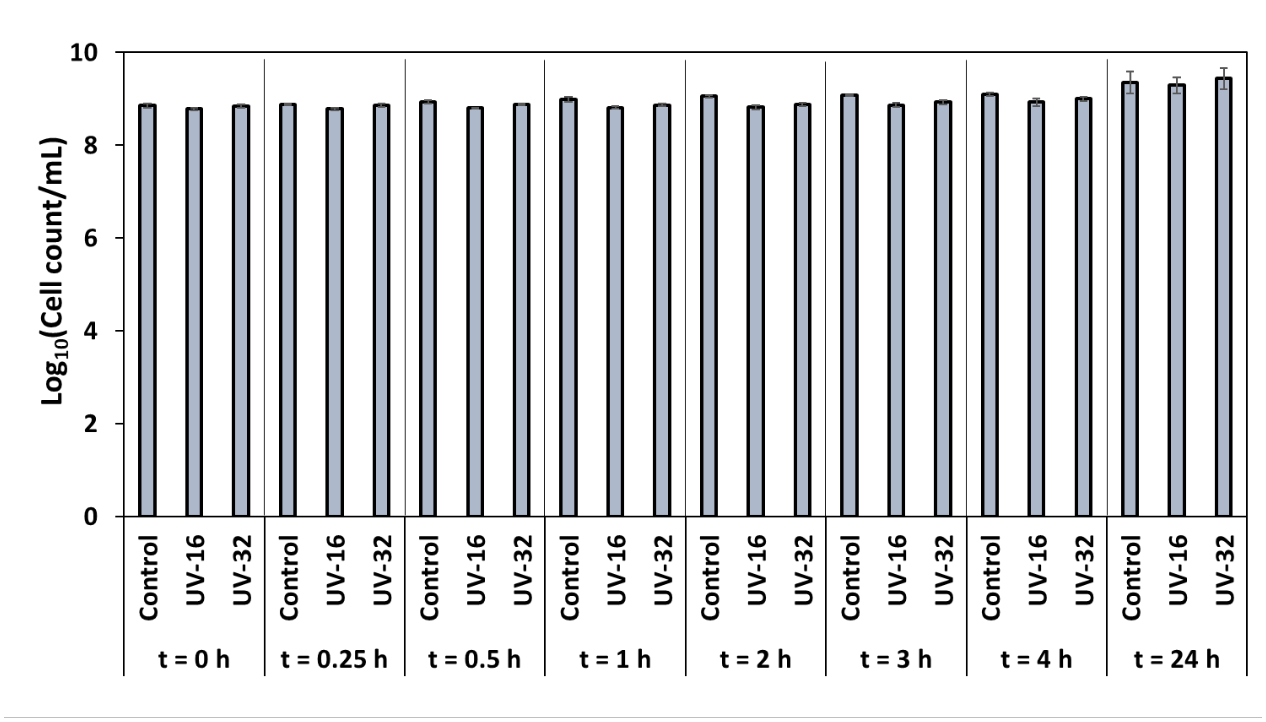

Fig. S7: Quantification of *E. coli* cell numbers during recovery following UV-B treatment. Cells were quantified by flow cytometry at the indicated recovery time points for untreated controls (Control), 16-min UV-B treatment (UV-16), and 32-min UV-B treatment (UV-32). Propidium iodide (PI) staining was used to gate live cells; however, under these conditions cells were not permeabilized and did not stain as dead after UV treatment. n = 4. Data corresponding to each time point represent mean value ± standard deviation.

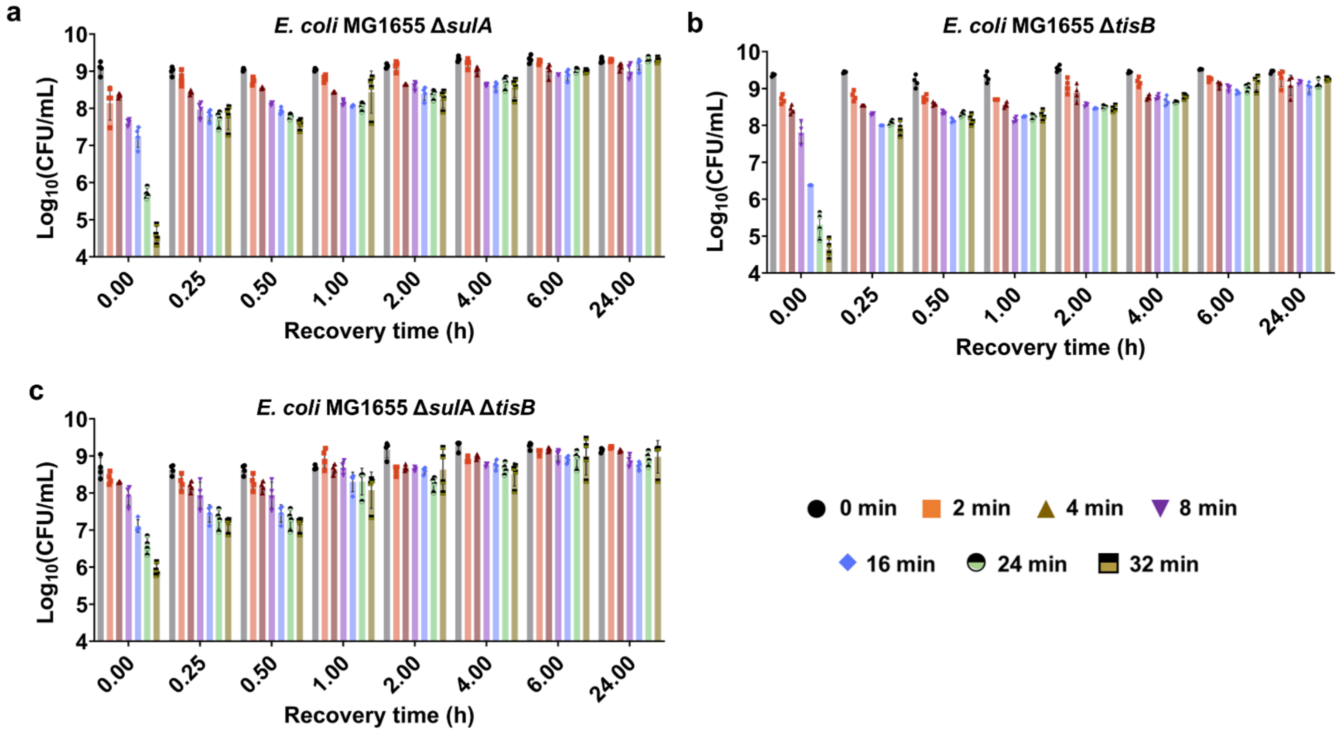

Fig. S8: Assessment of cell culturability over 24 hours in *E. coli* MG1655 strains lacking SulA and TisB. Mid-exponential-phase *E. coli* MG1655 Δ*sulA*, Δ*tisB*, and Δ*sulA*Δ*tisB* cells were exposed to UV-B light for 0, 2, 4, 8, 16, 24, and 32 minutes, followed by a 24-h recovery period. At designated time points (0, 0.25, 0.5, 1, 2, 4, 6, and 24 h), samples were collected and plated to assess CFU. n = 4. Data represents the mean ± standard deviation at each time point.

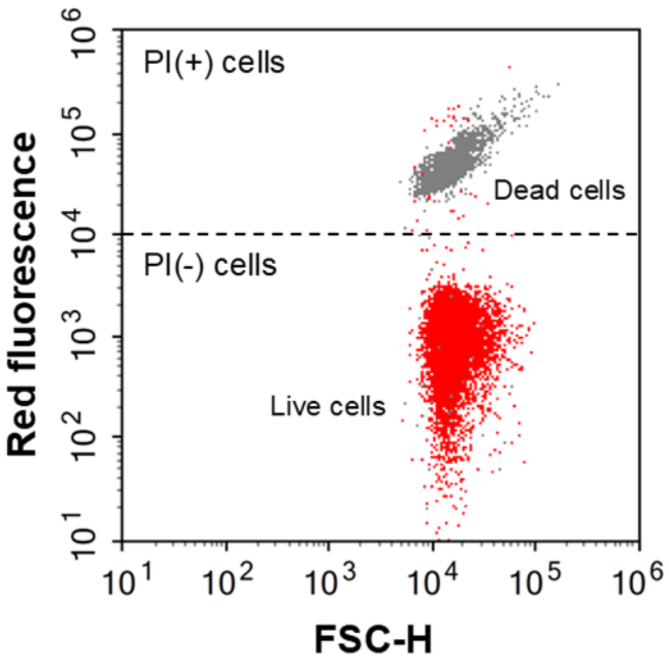

Fig. S9: PI staining of live and dead cells. Live cells (Red) and ethanol-treated dead cells (70% v/v, Grey) were stained with PI and analyzed by flow cytometry to assess live and dead cell populations. The figure shows a representative flow cytometry diagram.

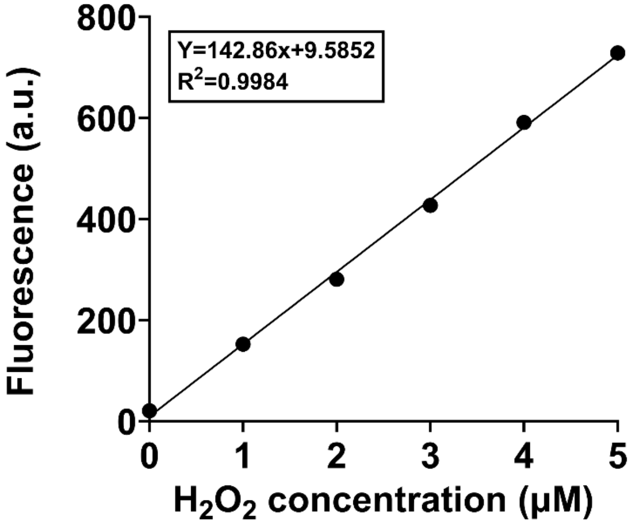

Fig. S10: Standard curve for H_2O_2 measurement using the Amplex Red Hydrogen Peroxide/Peroxidase Assay Kit. The standard curve was generated using a 20 mM Hydrogen Peroxide (H_2O_2) working solution, prepared by dissolving 3.0% (0.88 M) H_2O_2 in 1X Reaction Buffer. This solution was then diluted in 1X Reaction Buffer to achieve H_2O_2 concentrations ranging from 0 to 10 µM, in 50 µL aliquots. To each microplate well containing the standards, 50 µL of Amplex® Red reagent/HRP working solution was added. After a 30-minute incubation, fluorescence was measured using a microplate reader with excitation in the range of 530–560 nm and emission detection at ~590 nm.

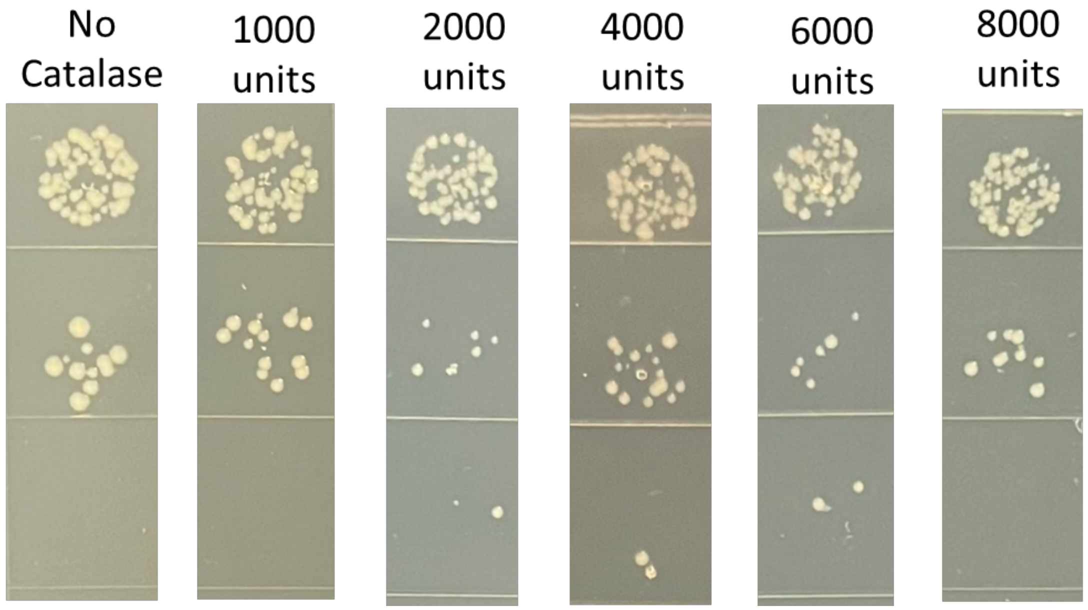

Fig. S11. Effect of catalase supplementation on recovery of CFU following prolonged UV-B exposure. To test whether oxidative stress on solid medium contributes to the reduced CFU levels observed immediately after prolonged UV-B exposure, LB agar plates were supplemented with increasing concentrations of catalase (0, 1,000, 2,000, 4,000, and 8,000 units per plate). Mid-exponential phase *E. coli* MG1655 cultures were exposed to 32 minutes of UV-B. Following treatment, 10 µl samples were serially diluted in 90 µl PBS, and 10 µl of each dilution was immediately plated onto catalase-supplemented and control LB agar plates. This dilution scheme allowed clear colonies to be observed in the first and second dilution spots on the agar. The resulting CFU values, after accounting for the dilution factor (~10^5 CFU/mL), were consistent with the t = 0 h data point for the 32-min UV-treated culture shown in Figure 1b of the main text. Catalase supplementation did not improve CFU recovery at any concentration tested compared to control, indicating that exogenous degradation of hydrogen peroxide on agar plates does not rescue the early CFU impairment observed after prolonged UV-B treatment.

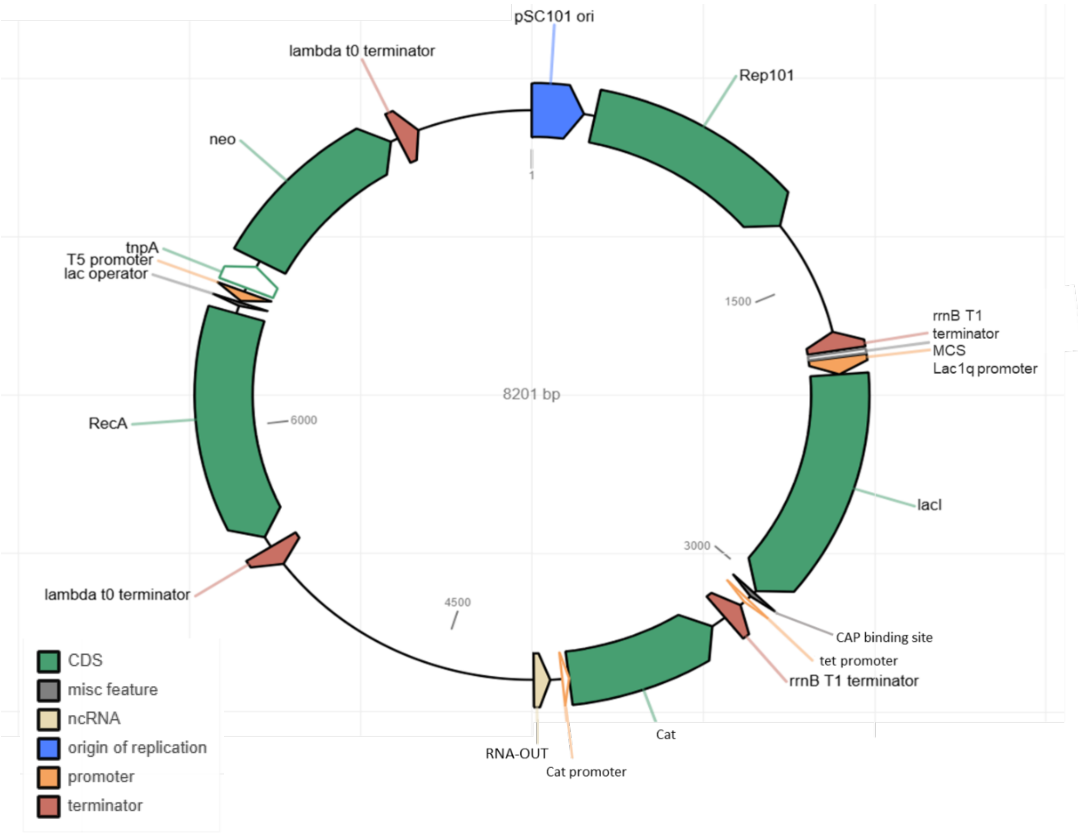

Fig. S12: Annotated plasmid map of the overexpression plasmid pUA66-*recA*. An IPTG-inducible *recA* overexpression plasmid was constructed by a commercial cloning service (Synbio Technologies, USA). The *recA* coding sequence from *E. coli* MG1655 was inserted downstream of a T5 promoter in the low-copy plasmid backbone pUA66, which includes a strong mutated *lacI* repressor for tight regulation and a kanamycin resistance marker. The plasmid was verified by sequencing (Plasmidsaurus, USA) and transformed into MG1655 Δ*recA* cells for inducible expression studies.

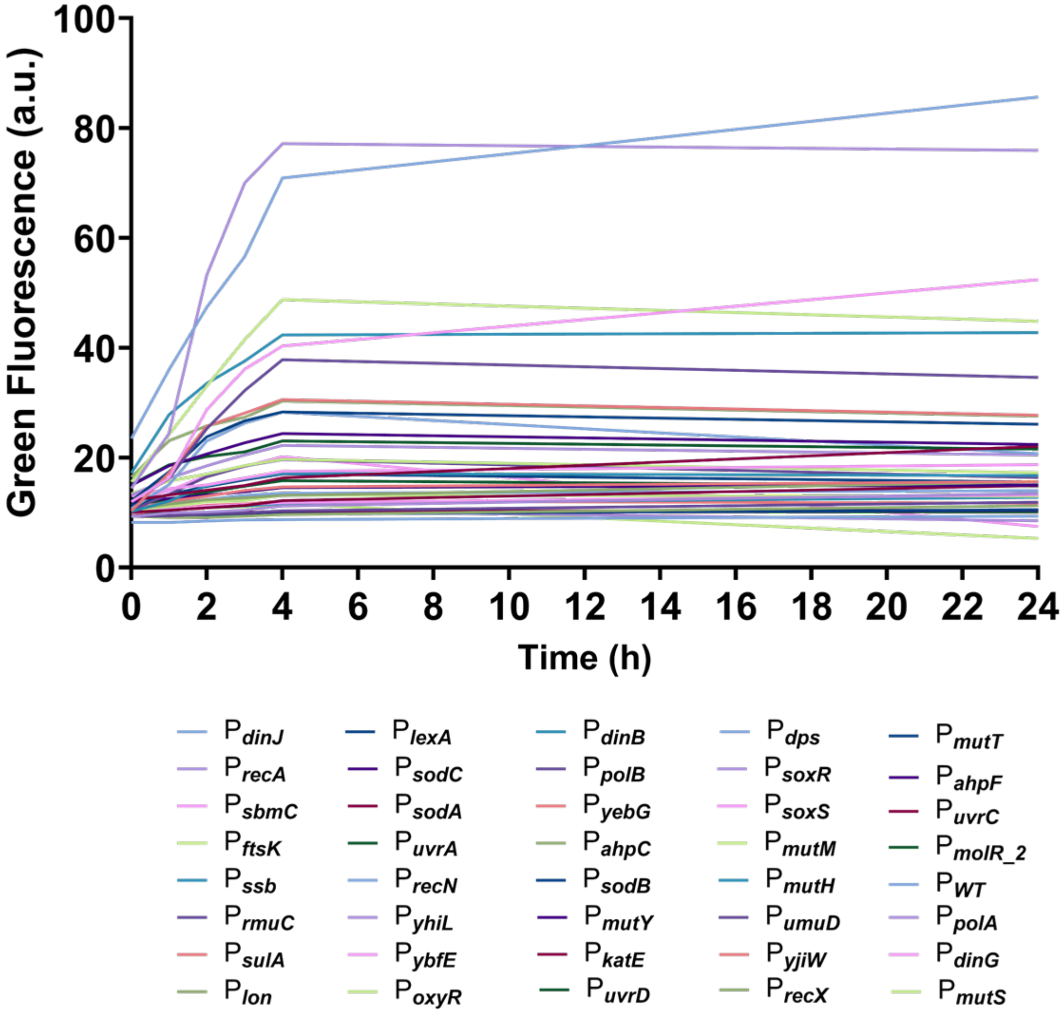

Fig. S13. Time-resolved GFP expression from promoter-GFP reporters following UV-B exposure. *E. coli* MG1655 cells carrying selected promoter-GFP reporters from the promoter library were exposed to 16 minutes of UV-B and allowed to recover. GFP levels were monitored over time using a plate reader to assess the temporal dynamics of UV-induced promoter activity. Because many SOS reporters exhibit relatively low activity and are difficult to quantify by plate reading, we used 24-hour, high-cell-density cultures to ensure reliable fluorescence measurements in this high-throughput format.

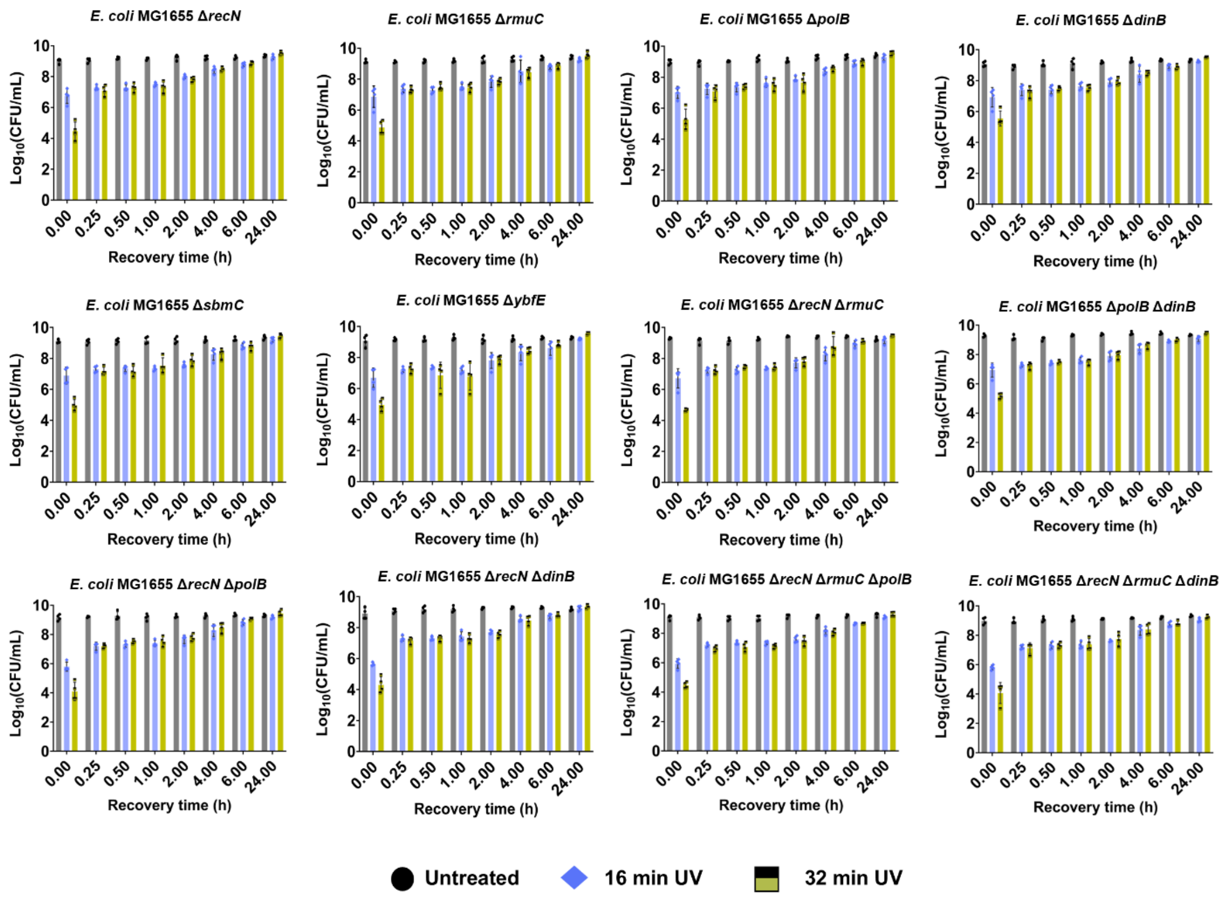

Fig. S14: Assessment of cell culturability over 24 hours in *E. coli* MG1655 strains lacking UV-upregulated genes identified in the promoter screening. Genes that were upregulated following UV exposure in the MG1655 promoter-reporter library were individually or combinatorially deleted, and UV-induced mutagenesis assays were performed in the resulting knockout strains. Mid-exponential phase cells were exposed to UV-B for 0, 16, or 32 minutes and allowed to recover for 24 hours. At specified recovery time points (0, 0.25, 0.5, 1, 2, 4, 6, and 24 h), samples were collected and plated to assess CFU. n = 4. Data represent mean ± standard deviation for each time point.

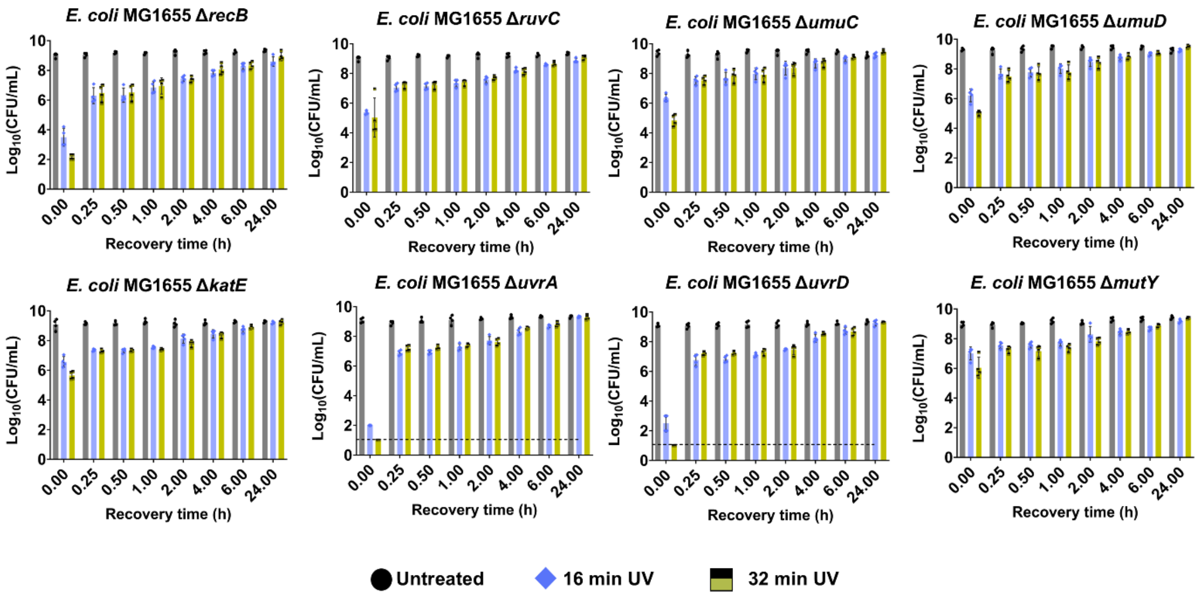

Fig. S15: Assessment of cell culturability over 24 hours in *E. coli* MG1655 strains lacking selected genes from knockout library screening. Selected genes identified in the *E. coli* BW25113 single-gene deletion screen were individually deleted in the MG1655 background, and UV-induced mutagenesis assays were performed. Mid-exponential phase cells were exposed to UV-B for 0, 16, or 32 minutes, followed by recovery for 24 hours. At designated time points (0, 0.25, 0.5, 1, 2, 4, 6, and 24 h), samples were collected and plated to assess CFU. n = 4. Data represent the mean ± standard deviation at each time point.

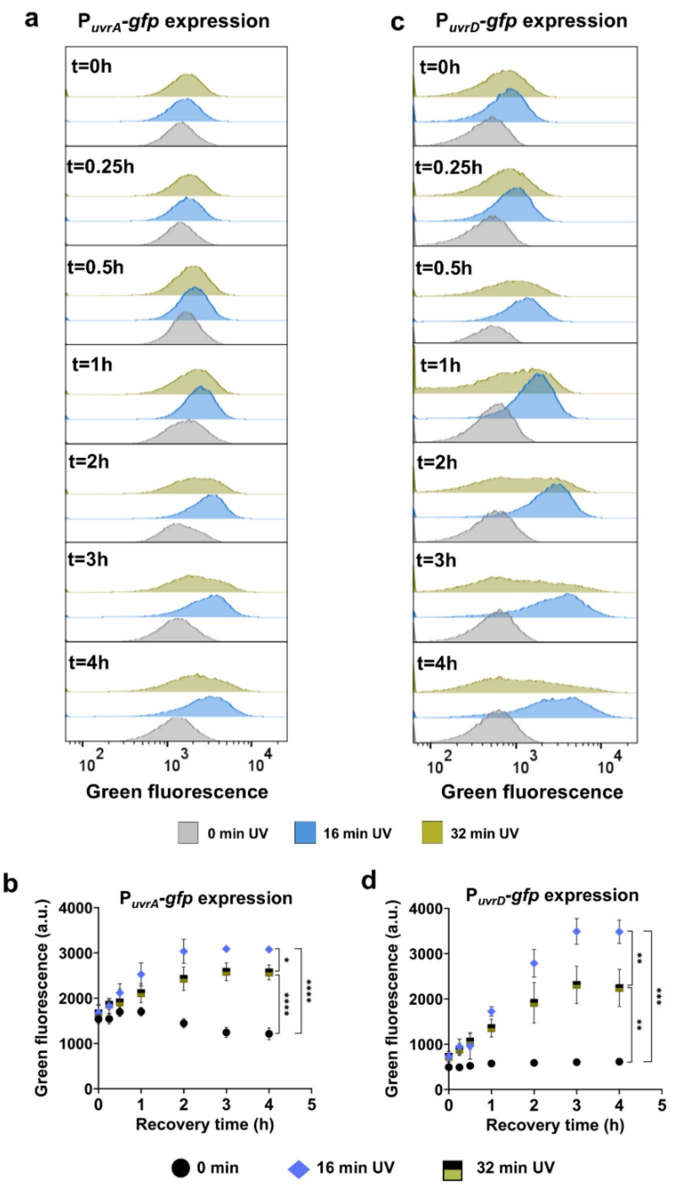

Fig. S16: Flow cytometry analysis of nucleotide excision repair genes followed by UV-treatment (a, b) The GFP profiles of UV-treated *E. coli* MG1655 cells harboring *pMSs201* P_{uvrA}-*gfp*, and (c, d) the GFP profiles of UV-treated *E. coli* MG1655 cells harboring *pMSs201* P_{uvrD}-*gfp* were assessed using flow cytometry after UV exposure (for the indicated time points during recovery). n=4. Statistical analysis was performed using RM two-way ANOVA with Greenhouse-Geisser correction and Tukey's multiple comparison test, where $*P < 0.05$, $**P < 0.01$, $***P < 0.001$, $****P < 0.0001$. Data corresponding to each time point represent mean value ± standard deviation.

Note: P_{recA}-*gfp* expression reached 10^5–10^6 (a.u.) (Fig. 3c), whereas P_{uvrA}-*gfp* and P_{uvrD}-*gfp* signals were only 10^3–10^4 (a.u.) following 16-min UV-B treatment. These low levels likely explain why their expression changes were not detectable with the lower sensitivity of plate reader assays.

Supplementary tables

Table S1: Mutations identified in the *rpoB* locus of rifampicin-resistant colonies following UV-B treatment

Sample Condition	Replicate	Genome Position	rpoB Position (nt)	Nucleotide Change	Codon Change	Amino Acid Change	Region
Untreated	Control	-	-	None	-	-	-
UV-B (16 min)	Sample 1	4,182,836	1592	C → T	TCC → TTC	Ser → Phe	RRDR (~507–533 aa)
UV-B (16 min)	Sample 2	4,182,836	1592	C → T	TCC → TTC	Ser → Phe	RRDR (~507–533 aa)
UV-B (16 min)	Sample 3	4,182,836	1592	C → T	TCC → TTC	Ser → Phe	RRDR (~507–533 aa)
UV-B (16 min)	Sample 4	4,182,836	1592	C → A	TCC → TAC	Ser → Tyr	RRDR (~507–533 aa)

Table S2: Promoter and single-deletion knockout libraries used in this study

Bacterial Strains	Source
E. coli K-12 MG1655 *pMSs201* P_{uvrA}-*gfp*	Promoter library from Horizon Discovery, Lafayette, CO, USA
E. coli K-12 MG1655 *pMSs201* P_{dps}-*gfp*	
E. coli K-12 MG1655 *pMSs201* P_{soxS}-*gfp*	
E. coli K-12 MG1655 *pMSs201* P_{recX}-*gfp*	
E. coli K-12 MG1655 *pMSs201* P_{molR}-*gfp*	
E. coli K-12 MG1655 *pMSs201* P_{polA}-*gfp*	
E. coli K-12 MG1655 *pMSs201* P_{dinG}-*gfp*	
E. coli K-12 MG1655 *pMSs201* P_{mutS}-*gfp*	
E. coli K-12 MG1655 *pMSs201* P_{mutH}-*gfp*	
E. coli K-12 MG1655 *pMSs201* P_{mutY}-*gfp*	
E. coli K-12 MG1655 *pMSs201* P_{ybfE}-*gfp*	
E. coli K-12 MG1655 *pMSs201* P_{sbmC}-*gfp*	
E. coli K-12 MG1655 *pMSs201* P_{mutM}-*gfp*	
E. coli K-12 MG1655 *pMSs201* P_{yjiW}-*gfp*	
E. coli K-12 MG1655 *pMSs201* P_{mutT}-*gfp*	
E. coli K-12 MG1655 *pMSs201* P_{dinB}-*gfp*	
E. coli K-12 MG1655 *pMSs201* P_{ftsK}-*gfp*	

E. coli K-12 MG1655 *pMSs201* P_{sodB}-*gfp*	
E. coli K-12 MG1655 *pMSs201* P_{recN}-*gfp*	
E. coli K-12 MG1655 *pMSs201* P_{rmuC}-*gfp*	
E. coli K-12 MG1655 *pMSs201* P_{dinJ}-*gfp*	
E. coli K-12 MG1655 *pMSs201* P_{yebG}-*gfp*	
E. coli K-12 MG1655 *pMSs201* P_{sulA}-*gfp*	
E. coli K-12 MG1655 *pMSs201* P_{soxR}-*gfp*	
E. coli K-12 MG1655 *pMSs201* P_{ahpC}-*gfp*	
E. coli K-12 MG1655 *pMSs201* P_{katE}-*gfp*	
E. coli K-12 MG1655 *pMSs201* P_{sodC}-*gfp*	
E. coli K-12 MG1655 *pMSs201* P_{ahpF}-*gfp*	
E. coli K-12 MG1655 *pMSs201* P_{yhiL}-*gfp*	
E. coli K-12 MG1655 *pMSs201* P_{uvrC}-*gfp*	
E. coli K-12 MG1655 *pMSs201* P_{oxyR}-*gfp*	
E. coli K-12 MG1655 *pMSs201* P_{polB}-*gfp*	
E. coli K-12 MG1655 *pMSs201* P_{uvrD}-*gfp*	
E. coli K-12 MG1655 *pMSs201* P_{sodA}-*gfp*	
E. coli K-12 MG1655 *pMSs201* P_{lexA}-*gfp*	
E. coli K-12 MG1655 *pMSs201* P_{recA}-*gfp*	
E. coli K-12 MG1655 *pMSs201* P_{lon}-*gfp*	
E. coli K-12 MG1655 *pMSs201* P_{umuD}-*gfp*	
E. coli K-12 MG1655 *pMSs201* P_{ssb}-*gfp*	
E. coli K-12 BW25113 Δ*fnr*	Keio collection, Cat# OEC4987
E. coli K-12 BW25113 Δ*soxR*	
E. coli K-12 BW25113 Δ*soxS*	
E. coli K-12 BW25113 Δ*umuD*	
E. coli K-12 BW25113 Δ*recA*	
E. coli K-12 BW25113 Δ*ruvB*	
E. coli K-12 BW25113 Δ*uvrD*	
E. coli K-12 BW25113 Δ*sodB*	
E. coli K-12 BW25113 Δ*sodC*	
E. coli K-12 BW25113 Δ*ahpF*	
E. coli K-12 BW25113 Δ*katG*	
E. coli K-12 BW25113 Δ*sodA*	
E. coli K-12 BW25113 Δ*ygjF*	
E. coli K-12 BW25113 Δ*polA*	
E. coli K-12 BW25113 Δ*ruvA*	
E. coli K-12 BW25113 Δ*ruvC*	
E. coli K-12 BW25113 Δ*umuC*	

E. coli K-12 BW25113 Δ*uvrA*	
E. coli K-12 BW25113 Δ*alkA*	
E. coli K-12 BW25113 Δ*ahpC*	
E. coli K-12 BW25113 Δ*sulA*	
E. coli K-12 BW25113 Δ*katE*	
E. coli K-12 BW25113 Δ*uvrB*	
E. coli K-12 BW25113 Δ*nei*	
E. coli K-12 BW25113 Δ*dinB*	
E. coli K-12 BW25113 Δ*recB*	
E. coli K-12 BW25113 Δ*nth*	
E. coli K-12 BW25113 Δ*uvrC*	
E. coli K-12 BW25113 Δ*fur*	
E. coli K-12 BW25113 Δ*ligB*	
E. coli K-12 BW25113 Δ*ung*	
E. coli K-12 BW25113 Δ*tag*	
E. coli K-12 BW25113 Δ*recN*	
E. coli K-12 BW25113 Δ*mutY*	
E. coli K-12 BW25113 Δ*ycaQ*	
E. coli K-12 BW25113 Δ*mutM*	
E. coli K-12 BW25113 Δ*recC*	
E. coli K-12 BW25113 Δ*polB*	
E. coli K-12 BW25113 Δ*recD*	
E. coli K-12 BW25113 Δ*oxyR*	
E. coli K-12 BW25113 Δ*mutS*	
E. coli K-12 BW25113 Δ*mutH*	
E. coli K-12 BW25113 Δ*mutL*	
E. coli K-12 BW25113 Δ*exoI*	
E. coli K-12 BW25113 Δ*phrB*	
E. coli K-12 BW25113 Δ*lacI*	

Table S3: Bacterial plasmids and strains used in this study

**Bacterial Strains**	**Source**
E. coli K-12 MG1655 pUA66 P*recA-gfp*	Gift from Dr. Mark P. Brynildsen
E. coli K-12 MG1655 pUA66-*gfp*	Gift from Dr. Mark P. Brynildsen
E. coli K-12 MG1655 Wild type	Gift from Dr. Mark P. Brynildsen
E. coli K-12 MG1655 Δ*sulA*	Previous study
E. coli K-12 MG1655 Δ*tisB*	Previous study
E. coli K-12 MG1655 Δ*recA*	Previous study

E. coli K-12 MG1655 Δ*sulA*Δ*tisB*	This study
E. coli K-12 MG1655 Δ*recN*	This study
E. coli K-12 MG1655 Δ*rmuC*	This study
E. coli K-12 MG1655 Δ*polB*	This study
E. coli K-12 MG1655 Δ*dinB*	This study
E. coli K-12 MG1655 Δ*sbmC*	This study
E. coli K-12 MG1655 Δ*ybfE*	This study
E. coli K-12 MG1655 Δ*polB*Δ*dinB*	This study
E. coli K-12 MG1655 Δ*recN*Δ*rmuC*	This study
E. coli K-12 MG1655 Δ*recN*Δ*polB*	This study
E. coli K-12 MG1655 Δ*recN*Δ*dinB*	This study
E. coli K-12 MG1655 Δ*recN*Δ*rmuC*Δ*polB*	This study
E. coli K-12 MG1655 Δ*recN*Δ*rmuC*Δ*dinB*	This study
E. coli K-12 MG1655 Δ*recB*	This study
E. coli K-12 MG1655 Δ*ruvC*	This study
E. coli K-12 MG1655 Δ*umuC*	This study
E. coli K-12 MG1655 Δ*umuD*	This study
E. coli K-12 MG1655 Δ*katE*	This study
E. coli K-12 MG1655 Δ*uvrA*	This study
E. coli K-12 MG1655 Δ*uvrD*	Previous study
E. coli K-12 MG1655 Δ*mutY*	This study
E. coli K-12 MG1655 Δ*recA*+pUA66-*recA*	This study
E. coli K-12 MG1655 Δ*recA*+ pUA66-E.V.	This study

Table S4: Oligonucleotides used to generate single and multi-deletion mutants

**Oligonucleotides to generate gene deletions**			
**Mutation**	**Forward Primer (5' to 3'):**	**Reverse Primer (5' to 3')**	**Source**
Δ*recN*::KAN^R	ACACAATAACAGTAATGG TTTTTCATACAGGAAAAC GACTGTGTAGGCTGGAGC TGCTTC	CGTTTTGCTGTTTACTC TGACCGTGAAGCAGGA AAAAAGTTTAACGGCT GACATGGGAAT	Integrated DNA Technologies, Inc.
Δ*rmuC*::KAN^R	CAGGAAATGCCTTTCCA ACTGGACGTTTGTACAG CACAATTCTATTTTGTG CGGGTAAGTTGTTGCGT CAGGAGGCGTTGTGTA GGCTGGAGCTGCTTC	TCAAAAAATTGTTCC AGAAGTGTAACAGAT TGGGCGTCGATGCCC TAGATTTCTACCCGG CTTAACTACTCCCAA TGGGTTAACGGCTGA CATGGGAAT	Integrated DNA Technologies, Inc.

Δ*polB*::KAN^R	CAGGCTATACTCAAGCC<br>TGGTTTTTTGATGGATT<br>TTCAGCGTGTAGGCTGG<br>AGCTGCTTC	TCACGCATCAAAATG<br>GTATCTGGCGAACTC<br>TTTTTTTTGCTTAACG<br>GCTGACATGGGAAT	Integrated DNA Technologies, Inc.
Δ*dinB*::KAN^R	ACGCGTTAAATGCTGA<br>ATCTTTACGCATTTCTC<br>AAACCCTGAAATCACT<br>GTATACTTTA<br>CCAGTGTTGAGAGGTG<br>AGCA<br>GTGTAGGCTGGAGCTG<br>CTTC	CCGATTTTTCAGCGA<br>GAATTCGATGCATAC<br>AGTGATACCCTCATA<br>ATAATGCACACCAGA<br>ATATACATAATAGTA<br>TACATTAACGGCTGA<br>CATGGGAAT	Integrated DNA Technologies, Inc.
Δ*sbmC*::KAN^R	CAACTATACTGTATATA<br>AAAACAGTATCAATGG<br>AGGCGTCGTGTAGGCT<br>GGAGCTGCTTC	AAAGAGTGGTCATCG<br>CGTTAACACACCGCC<br>CTGAGATGAATTAAC<br>GGCTGACATGGGAA	Integrated DNA Technologies, Inc.
Δ*ybfE*::KAN^R	AGAGGCCTGGCTGATT<br>GTTTCCCCCGAAGTCAC<br>CAAGATCGTGTAGGCT<br>GGAGCTGCTTC	ACGAGCAGGACTGCA<br>CACTGTGCTACATGA<br>AAGTGGAAATTTAAC<br>GGCTGACATGGGAAT	Integrated DNA Technologies, Inc.
Δ*recB*::KAN^R	AGCGCGTTGCAGCAAA<br>CAATGCCCCTGATGAGT<br>GAAAAGAGTGTAGGCT<br>GGAGCTGCTTC	GCGGGCGTAGCTGTT<br>TGTGCTCCACAGCTT<br>CCAGTAATTGCTTTT<br>GCAATTTCATTAACG<br>GCTGACATGGGAAT	Integrated DNA Technologies, Inc.
Δ*ruvC*::KAN^R	CTCTGATGAGGCCTGCT<br>AAACAGCAAAACGGAG<br>ACGCGTGGTGTAGGCT<br>GGAGCTGCTTC	GTGGAAACGCCTCAG<br>CCGGAACTGACCGAG<br>GCGGTATAACTTAAC<br>GGCTGACATGGGAAT	Integrated DNA Technologies, Inc.
Δ*umuC*::KAN^R	TCTTTGGTGTGGTGATC<br>CACGTCGTTAAGGCGAT<br>GCGCTGGTGTAGGCTG<br>GAGCTGCTTC	TCGGCGCTCCTGCGG<br>GAGCGCTTTTTTCCTG<br>CCGCTATATTTAACG<br>GCTGACATGGGAA	Integrated DNA Technologies, Inc.
Δ*umuD*::KAN^R	GAACAGACTACTGTAT<br>ATAAAAACAGTATAAC<br>TTCAGGCAGATTATTGT<br>GTAGGCTGGAGCTGCTT<br>C	CAGCTGGCATAAAAC<br>GCGTTTACATCACAG<br>AGGGCAAACATTTAA<br>CGGCTGACATGGGAA<br>T	Integrated DNA Technologies, Inc.
Δ*katE*::KAN^R	ACAGCGGCCCTTTCAGT<br>AATAAATTAAGGAGAC<br>GAGTTCAGTGTAGGCTG<br>GAGCTGCTTC	ATGTAAATCATTTGA<br>GGCGGCGCAATTGCG<br>CCGCCTCCCATTAAC<br>GGCTGACATGGGAA	Integrated DNA Technologies, Inc.
Δ*uvrA*::KAN^R	ATGCCACCGGGCAAAA<br>AAGCGTTTAATCCGGG<br>AAAGGTGAGTGTAGGC<br>TGGAGCTGCTTC	CTCTGAAAGGAAAAG<br>GCCGCTCAGAAAGCG<br>GCCTTAACGATTAAC<br>GGCTGACATGGGAA	Integrated DNA Technologies, Inc.

Δ*mutY*::KAN^R	TGCTGCAATCTTGCCCCCAACAACAGTGAATTCGGTGACCGTGTAGGCTGGAGCTGCTTC	TCGTTCTGCTCATAAATCATCCTCTTTATCGACTCACGCGTTAACGGCTGACATGGGAAT	Integrated DNA Technologies, Inc.
Δ*phrB*::KAN^R (Trial 1)	CTTTGGCCGCGTGTGATTAACTTGCGCCATTCAGGAGTTTTGTGTAGGCTGGAGCTGCTTC	CAGACGCGTCAGGCAATCGAGCCCAGATGCCGGATGCGGCTTAACGGCTGACATGGGAAT	Integrated DNA Technologies, Inc.
Δ*phrB*::KAN^R (Trial 2)	AGGGCAGCGTTATTTCGAACTTTGGCCGCGTGTGATTAACTTGCGCCATTCAGGAGTTTTGTGTAGGCTGGAGCTGCTTC	CTGTTCGGTCGCTAATCCATTCGGCGCTCCTGCGGGAGCGCTTTTTTCCTGCCGCTATATTTAACGGCTGACATGGGAAT	Integrated DNA Technologies, Inc.

Oligonucleotides to verify gene deletions

Mutation	External Forward Primer (5' to 3')	External Reverse Primer (5' to 3')	Internal Forward Primer (5' to 3')	Internal Reverse Primer (5' to 3')	Source
Δ*recN*::KAN^R	GATTCGTCGCTGTGATTACCATC	GCTCTTCGTCCAGATCATCCT	GCACAACTGACCATCAGCAA	CAGTTGCTGCTGTTCTTCCAGTAG	Integrated DNA Technologies, Inc.
Δ*rmuC*::KAN^R	AAAGCCATGCGGTGAAAATC	GCTCTTCGTCCAGATCATCCT	GTTATTGCGTTGGTGGGTGT	GGGTTCAACGGGAATAAACA	Integrated DNA Technologies, Inc.
Δ*polB*::KAN^R	GTCAGTTAGCGCCGCAGTTA	GCTCTTCGTCCAGATCATCCT	GCGCAGGCAGGTTTTATCTT	GGTTTATCTTCGGCGAAACG	Integrated DNA Technologies, Inc.
Δ*dinB*::KAN^R	GACCAAAAGTGCGTCCGATA	GCTCTTCGTCCAGATCATCCT	GTGGATATGGACTGCTTTTTCG	GGCGTTCATCCCAGGTTTTA	Integrated DNA Technologies, Inc.
Δ*sbmC*::KAN^R	GGTTACCCTGGAATCTGTCG	GCTCTTCGTCCAGATCATCCT	GAAGAGAAACGTACCGTTGCAG	GCTGCACCGCAACATACATT	Integrated DNA Technologies, Inc.
Δ*ybfE*::KAN^R	TACGCGCTATCCGTCGCTAT	GCTCTTCGTCCAGATCATCCT	AACAAACGGACCGTACGACA	GCCAGTTGCTGCATTAACATCT	Integrated DNA Technologies, Inc.
Δ*recB*::KAN^R	TTCTTCCATCAGGCGGTGGT	GCTCTTCGTCCAGATCATCCT	ATCCACTGTACGAACGCCTG	AAATTCGGTACTGCTGGGGG	Integrated DNA

					Technologies, Inc.
Δ*ruvC*::KAN^R	GAAACCG CACCGAA ACTGAT	GCTCTTC GTCCAGA TCATCCT	GGCTATTA TTCTCGGC ATTGATCC	AACGTGG CAGTGGG TGATAG	Integrated DNA Technologies, Inc.
Δ*umuC*::KAN^R	CTGTTGAC GGCGAGT TTACG	GCTCTTC GTCCAGA TCATCCT	AGCTGTGA GACGGTGT TTCG	GATCGCG TAGCAGC GTTAAT	Integrated DNA Technologies, Inc.
Δ*umuD*::KAN^R	TTTTCATT TCTGCTGG ATGAGC	GCTCTTC GTCCAGA TCATCCT	TCTTGTTC AGTGTGGC TTTCC	TGGATCA CCACACC AAAGAC A	Integrated DNA Technologies, Inc.
Δ*katE*::KAN^R	GTTTAGCC GATTTAGC CCCTG	GCTCTTC GTCCAGA TCATCCT	ATCAGCCG CTCACGGT TATT	TATGGTA AGGGCA GGTCGGA	Integrated DNA Technologies, Inc.
Δ*uvrA*::KAN^R	F_Ex_sodA: CACAACA CTCCGGGT AATGC	GCTCTTC GTCCAGA TCATCCT	TGAATCCC TTTCCGCC TACG	ACGGATG ACGACGA ATGGAG	Integrated DNA Technologies, Inc.
Δ*mutY*::KAN^R	CAAATTCC GGTGAAA TGACG	GCTCTTC GTCCAGA TCATCCT	GCCCAGGT TCTGGACT GGTA	CGACACG GGAAGCC ACATAG	Integrated DNA Technologies, Inc.
Δ*phrB*::KAN^R (Trial 1)	TCCTGACG CCTGGCTT TCAG	GCTCTTC GTCCAGA TCATCCT	ATATCGCT ACACCACG CCAG	AACAAGC GATGCAA GCACTG	Integrated DNA Technologies, Inc.
Δ*phrB*::KAN^R (Trial 2)	TTCCACAG AACGCCA GCAGC	GCTCTTC GTCCAGA TCATCCT	CCAGTGGG CGACGCAT AACA	AGCGGCA TCAACAA TCGGGT	Integrated DNA Technologies, Inc.
